# Supplementary material for: Switchable narrow nonlocal conducting polymer plasmonics
Source: Nat Commun. 2025 May 21;16:4484. doi: 10.1038/s41467-025-59764-5 (PMC12095564; doi:10.1038/s41467-025-59764-5)
Supplement: Supplementary file 1 — Supplementary Information [file 41467_2025_59764_MOESM1_ESM.pdf]

Supplementary Information

Switchable Narrow Nonlocal Conducting Polymer Plasmonics

Dongqing Lin[1], Yulong Duan[1], Pravallika Bandaru[1], Pengli Li[1], Mohammad Shaad Ansari[1], Alexander Yu. Polyakov [1], Janna Wilhelmsen[1], Magnus P. Jonsson[1,]*

[1]Laboratory of Organic Electronics, Department of Science and Technology (ITN), Linköping University, Norrköping, SE-60174, Sweden

Supplementary Note 1. Permittivity and matching conditions of collective lattice resonance (CLR) in square arrays

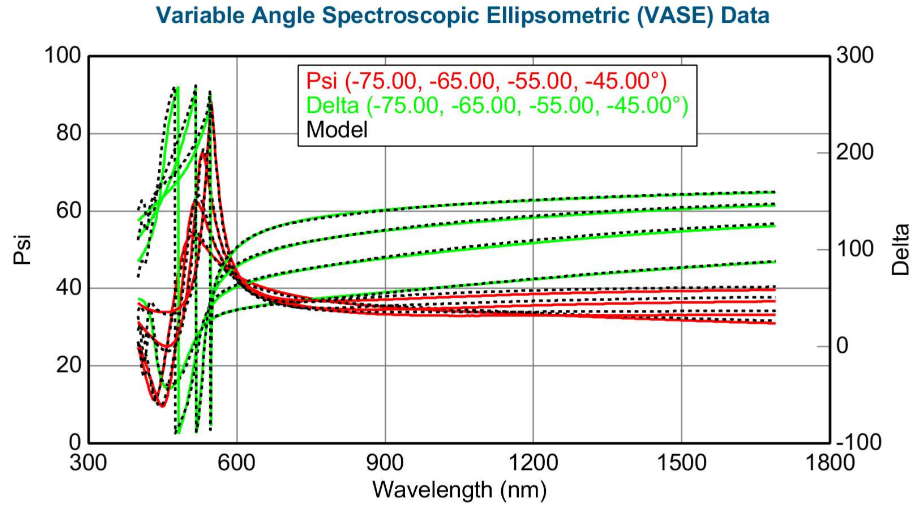

Supplementary Fig. 1 | Spectroscopic ellipsometry data (ranging from 210 nm to 1690 nm) for a PEDOT film (acid-treated PEDOT:ToS, with thickness of 200 nm). These raw data and the fitting were processed using the VASE software. The Psi (ψ, marked in the red line) and Delta (Δ, marked in the green line) were acquired at four angles (45°, 55°, 65°, and, 75°). The black dashed lines are the best fitting data by using the Drude-Lorents model.

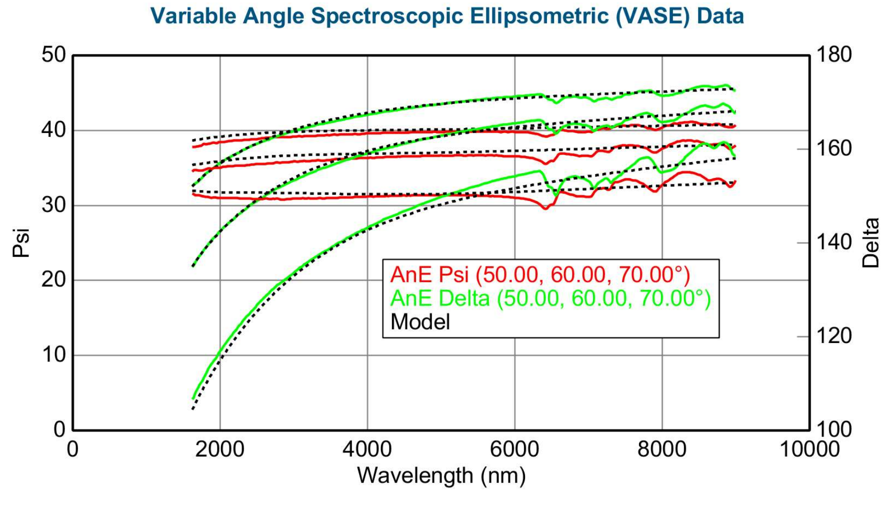

Supplementary Fig. 2 | Spectroscopic ellipsometry data (ranging from 1690 nm to 9000 nm) for a PEDOT film (acid-treated PEDOT:ToS, with thickness of 200 nm). These raw data and the fitting processing are based on the VASE software. The Psi (ψ, marked in the red line) and Delta (Δ, marked in the green line) are achieved with three angles (50°, 60°, and, 70°). The black dashed lines are shown in the best fitting data by using the Drude-Lorentz model.

The Drude-Lorentz equation for fitting permittivities[1] is described as:

$$\varepsilon(\omega) = \varepsilon_\infty - \frac{\omega_p^2}{\omega^2 + i\omega\gamma} - \sum_j \frac{A_j}{\omega^2 - \omega_j^2 + i\omega\gamma_j} \tag{1}$$

where ω is the angular frequency, ε_∞ is the permittivity at infinitely high frequency (beyond the measurement range), γ is the momentum-averaged broadening (related to the damping relaxation), i is the imaginary unit, ω_p is the plasma frequency. A_j, ω_j and γ_j are amplitude, resonance angular frequency, and broadening (or damping relaxation part) of the j-th Lorentz oscillator, respectively. The Drude part has a similar mathematic type of Lorentz oscillator, although with $\omega_j = 0$.

Supplementary Table 1: Oscillators for oxidized state along the in-plane direction

$\varepsilon_\infty = 2.310$			
Oscillator No. (j th)	Frequency ω_j (eV)	Broadening γ_j (eV)	Amplitude A_j (eV^2)
Drude	0	0.320	5.280
1	3.049	0.125	0.661
2	1.680	0.9827	1.242

Supplementary Table 2: Oscillators for oxidized state along the out-of-plane direction

$\varepsilon_\infty = 1.798$			
Oscillator No. (j th)	Frequency ω_j (eV)	Broadening γ_j (eV)	Amplitude A_j (eV^2)
Drude	0	0.329	0.128
1	8.000	3.932	6.291
2	4.532	0.604	0.125
3	0.711	1.050	0.248

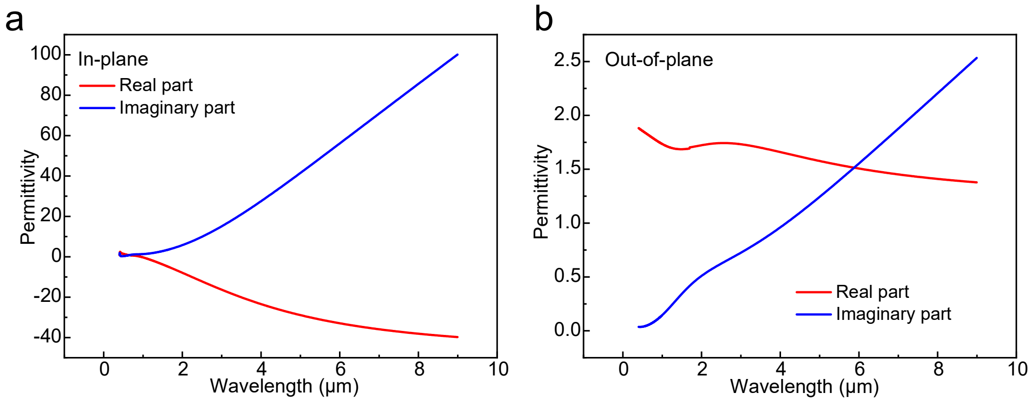

Supplementary Fig. 3 | Permittivity of acid-treated PEDOT:ToS (film thickness of 200 nm). These data were obtained from the calculation of Drude-Lorentz model by fitting the data of spectroscopic ellipsometry above. (a) Along the in-plane direction. (b) Along the out-of-plane direction.

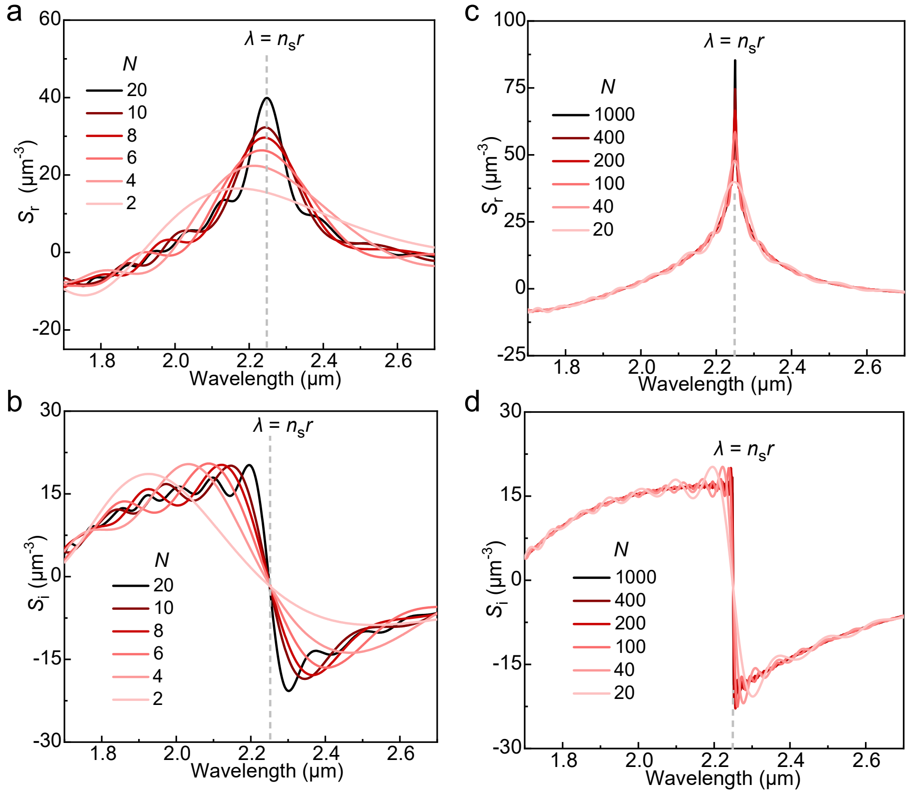

Supplementary Fig. 4 | Array factors (*S*) dependent on the number of other nanoantennas (*N*) along one side of a square-shaped lattice. The conditions including the periodicity r = 1.5 μm and the refractive index n_s = 1.5 in wave vector are used for these calculations. (a) (b) The real part (S_r) and imaginary part (S_i) of array factor curves, respectively, when N ranges from 2 to 20. (c) (d) S_r and S_i upon N = 20-1000, respectively. The wavelength ranges for all S curves are around $\lambda = n_s r$ = 2.25 μm.

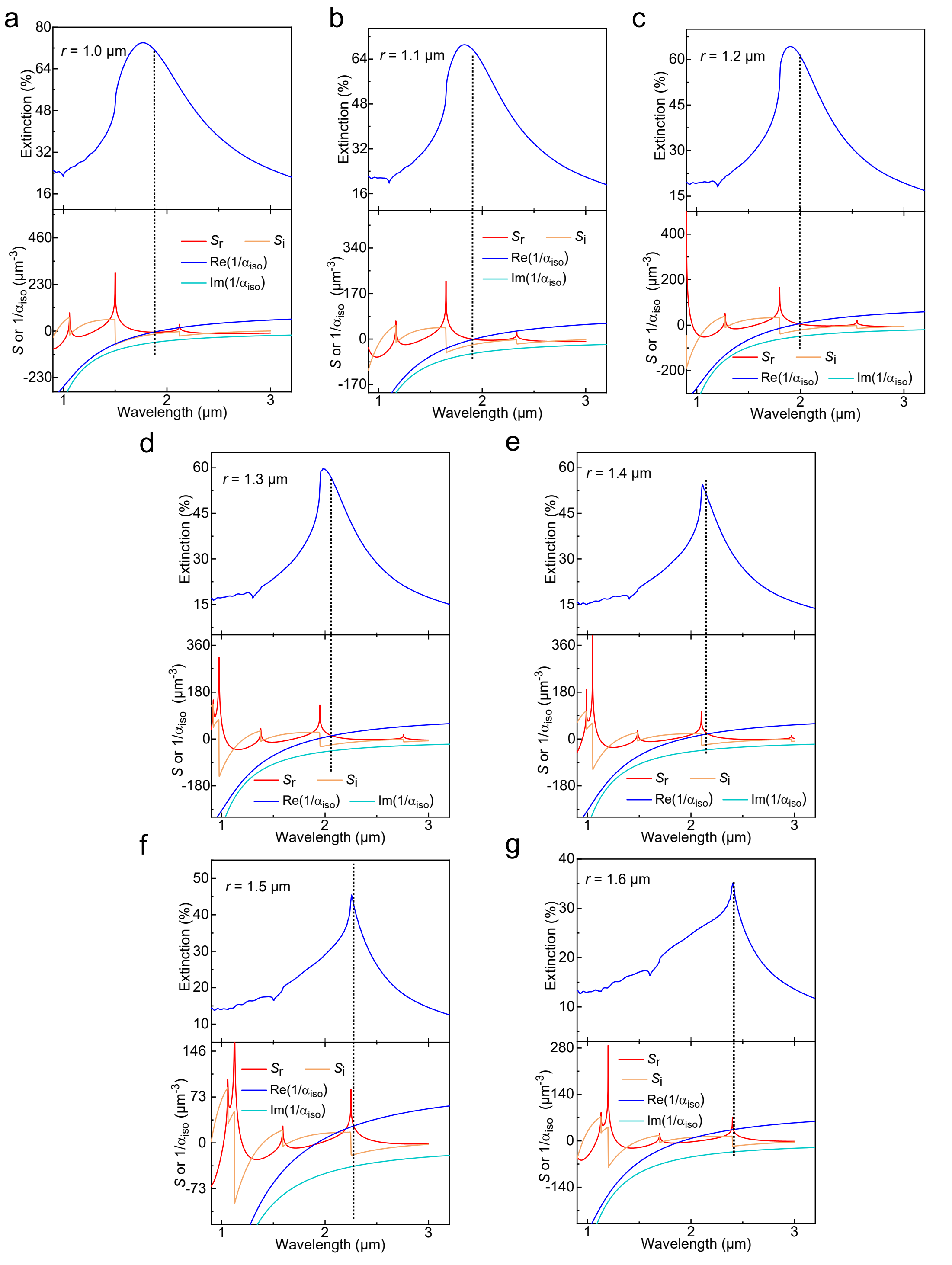

Supplementary Fig. 5 | Analysis of array factors and extinction spectra for square arrays with different periodic distances *r* = 1.0-1.6 μm, based on acid-treated PEDOT:ToS. The extinction spectra are were obtained by FDTD simulations. The diameter of the nanodisks was set to 0.52 μm, and the height of the nanodisks was set to 0.2 μm.

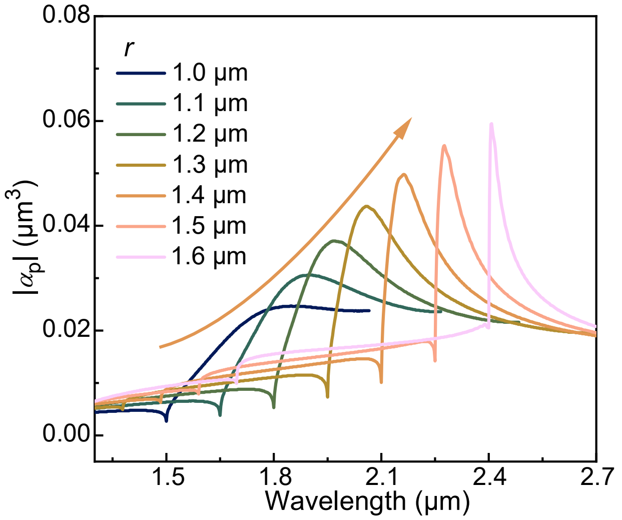

Supplementary Fig. 6 | Absolute periodic polarizability $|\alpha_p|$ of square-shaped arrays with different periodic distances *r* = 1.0-1.6 μm, based on acid-treated PEDOT:ToS nanoantennas with 0.52 μm diameter and 0.2 μm height. The peaks correspond to the surface plasmonic resonance based on the coupling interactions along the square-edges.

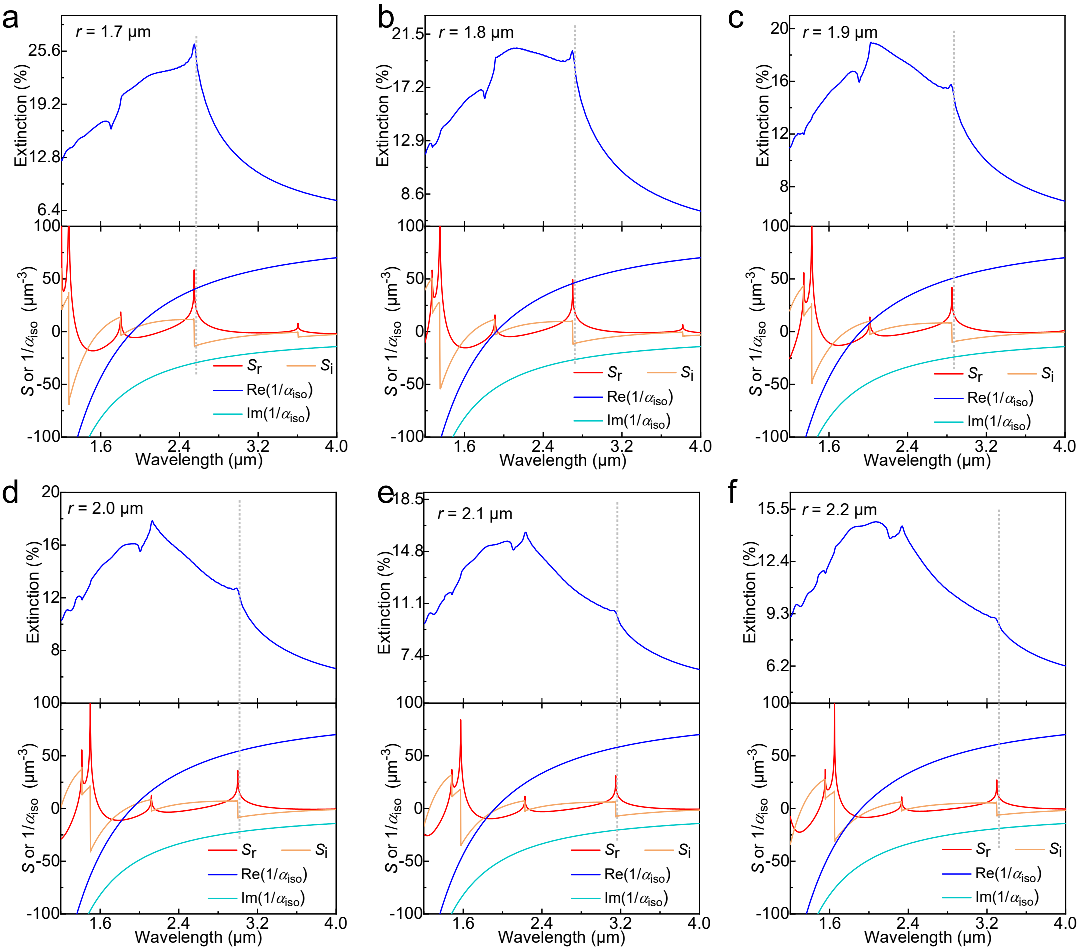

Supplementary Fig. 7 | Analysis of array factors and extinction spectra for square arrays with extended periodic distances *r* = 1.7-2.2 μm, based on acid-treated PEDOT:ToS. The extinction spectra are were obtained by FDTD simulations. The diameter of the nanodisks was set to 0.52 μm, and the height of the nanodisks was set to 0.2 μm.

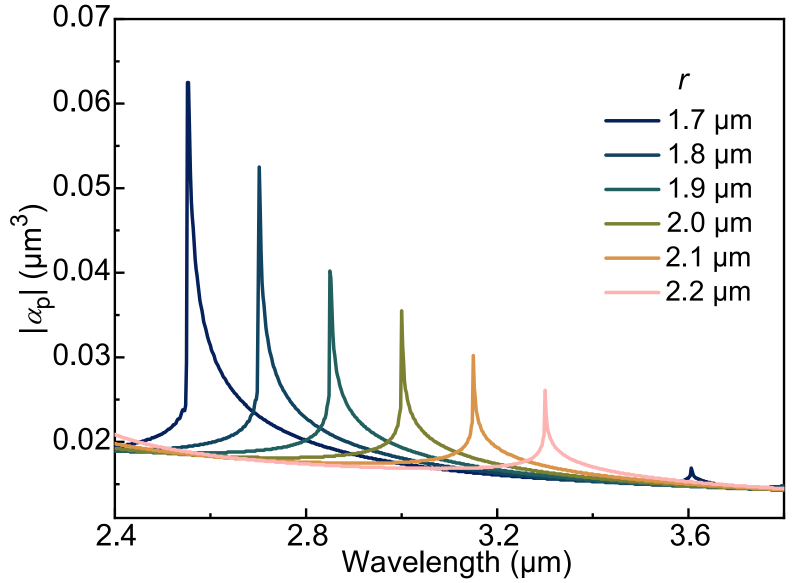

Supplementary Fig. 8 | Absolute periodic polarizability $|\alpha_p|$ of square-shaped arrays with extended periodic distances *r* = 1.7-2.2 μm, based on acid-treated PEDOT:ToS nanoantennas with 0.52 μm diameter and 0.2 μm height.

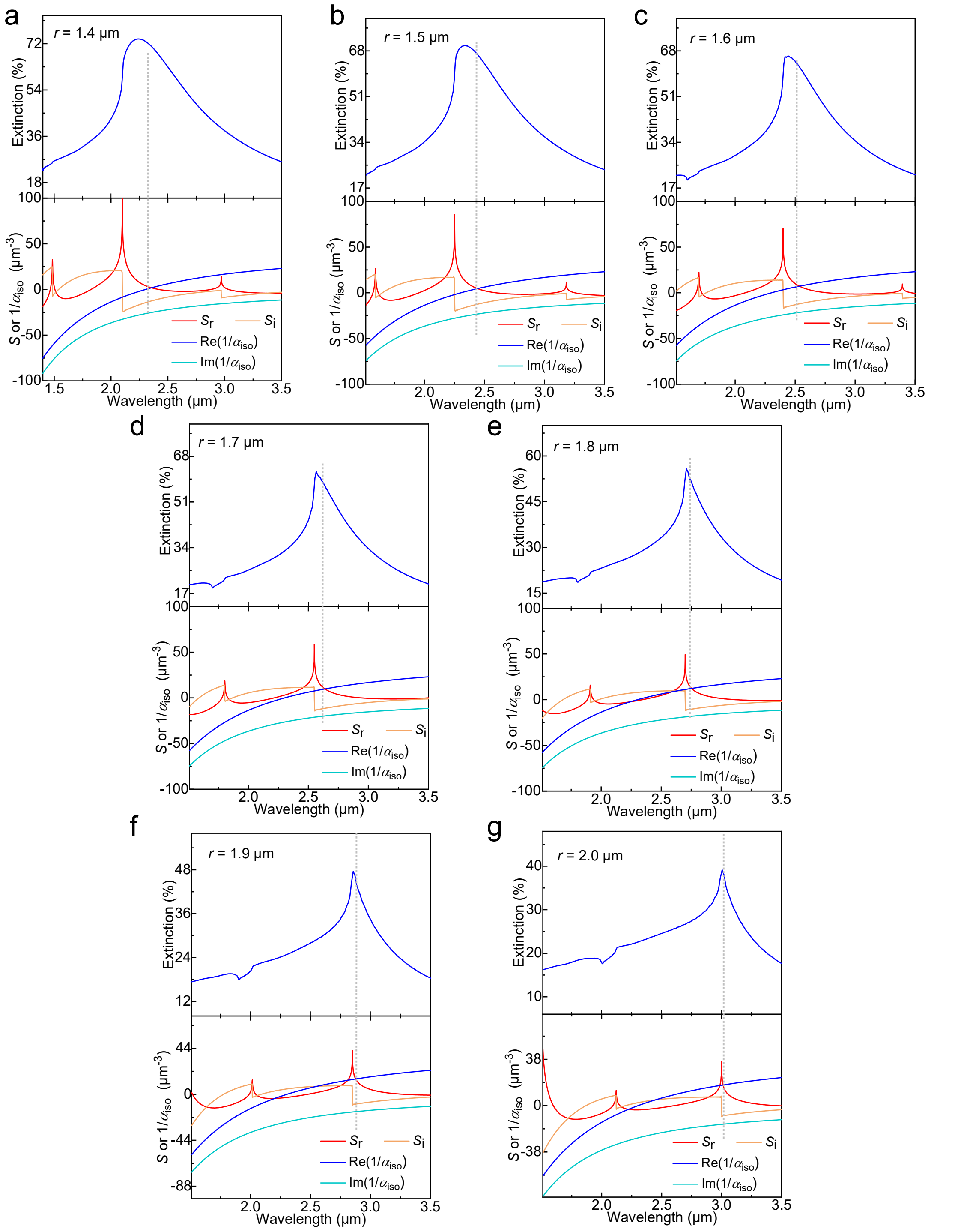

Supplementary Fig. 9 | Analysis of array factors and extinction spectra for square arrays with different periodic distances *r* = 1.4-2.0 μm, based on acid-treated PEDOT:ToS. The extinction spectra were obtained by FDTD simulations. The diameter of the nanodisks was set to 0.70 μm, and the height of the nanodisks was set to 0.2 μm.

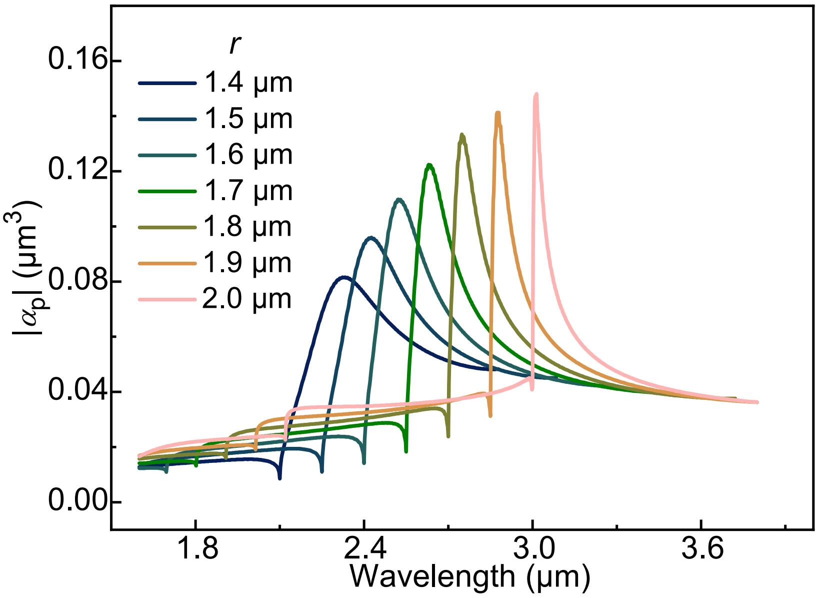

Supplementary Fig. 10 | Absolute periodic polarizability $|\alpha_p|$ of square-shaped arrays with different periodic distances r = 1.4-2.0 μm, based on acid-treated PEDOT:ToS nanoantennas with 0.70 μm diameter and 0.2 μm height.

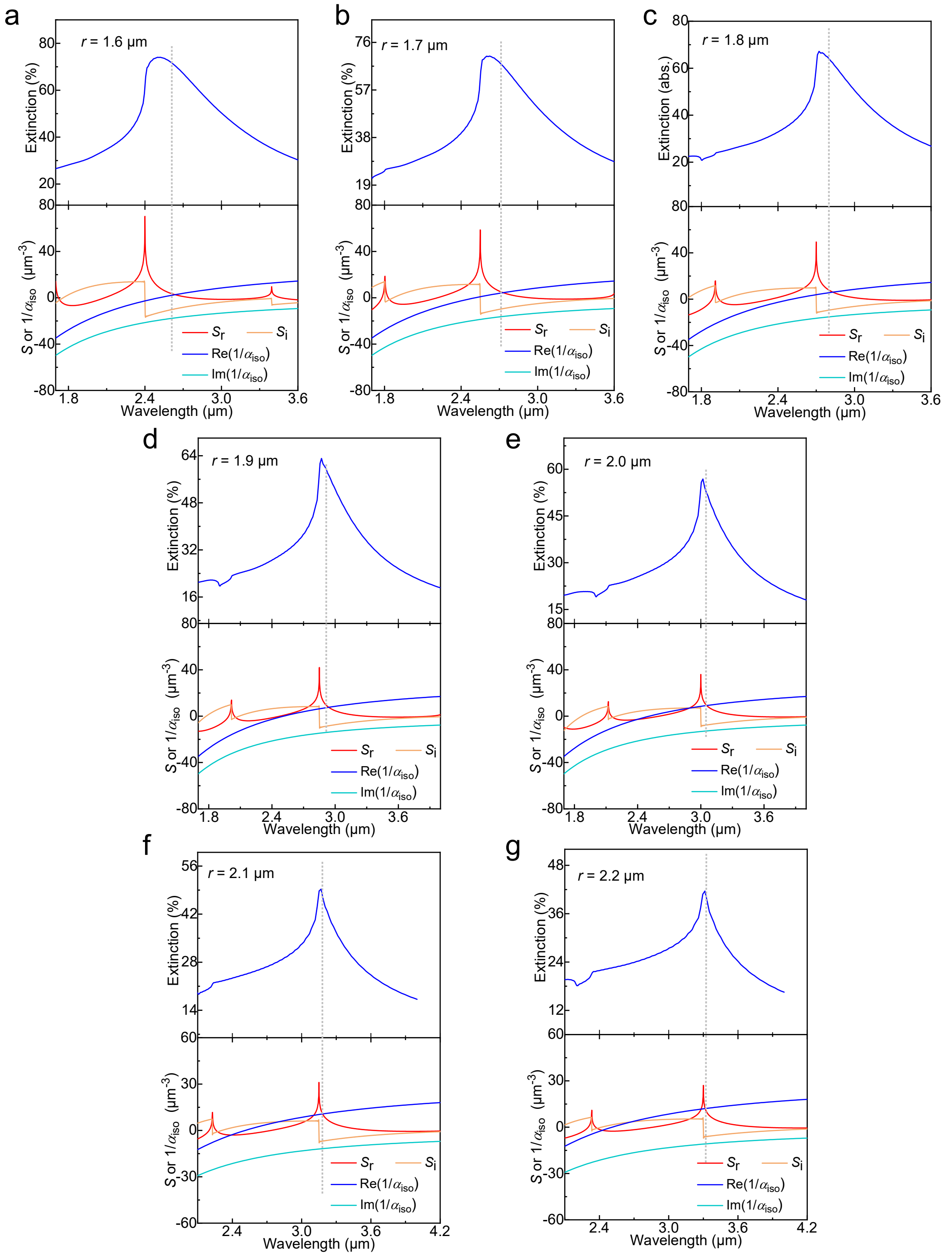

Supplementary Fig. 11 | Analysis of array factors and extinction spectra for square arrays with different periodic distances *r* = 1.6-2.2 μm, based on acid-treated PEDOT:ToS. The extinction spectra were obtained by FDTD simulations. The diameter of the nanodisks was set to 0.80 μm, and the height of the nanodisks was set to 0.2 μm.

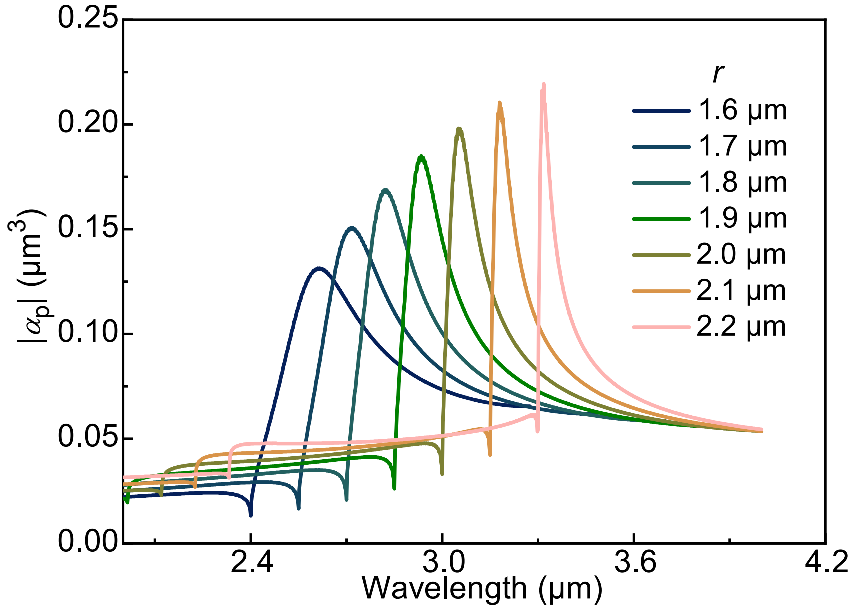

Supplementary Fig. 12 | Absolute periodic polarizability $|\alpha_p|$ of square-shaped arrays with different periodic distances *r* = 1.6-2.2 μm, based on acid-treated PEDOT:ToS nanoantennas with 0.80 μm diameter and 0.2 μm height.

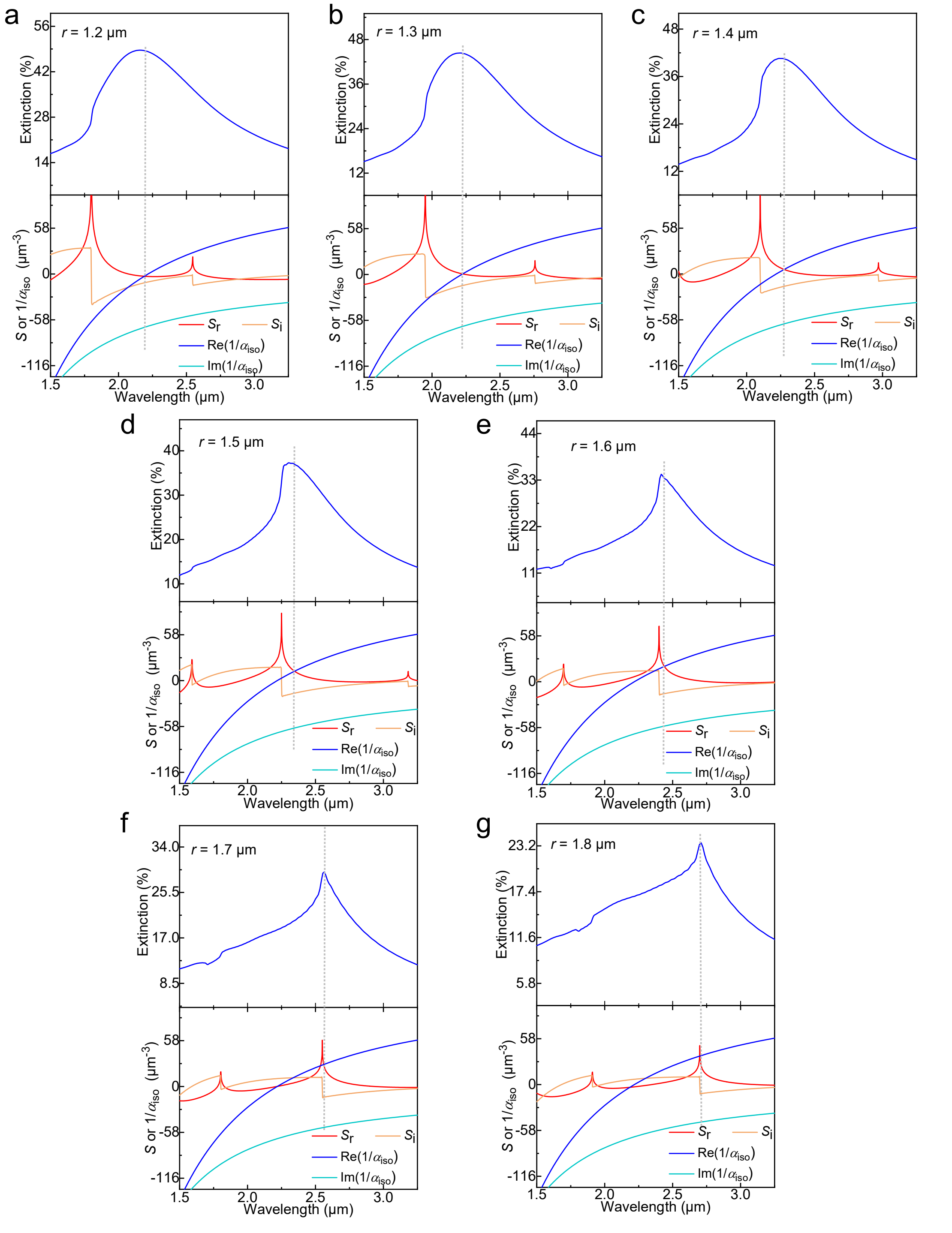

Supplementary Fig. 13 | Analysis of array factors and extinction spectra for square arrays with different periodic distances *r* = 1.2-1.8 μm, based on acid-treated PEDOT:ToS. The extinction spectra were obtained by FDTD simulations. The diameter of the nanodisks was set to 0.52 μm, and the height of the nanodisks was set to 0.1 μm.

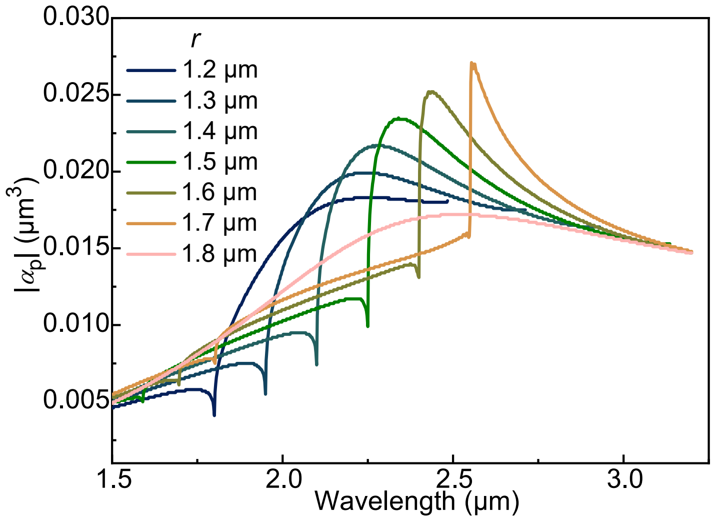

Supplementary Fig. 14 | Absolute periodic polarizability |α_p| of square-shaped arrays with different periodic distances r = 1.2-1.8 μm, based on acid-treated PEDOT:ToS nanoantennas with 0.52 μm diameter and 0.1 μm height.

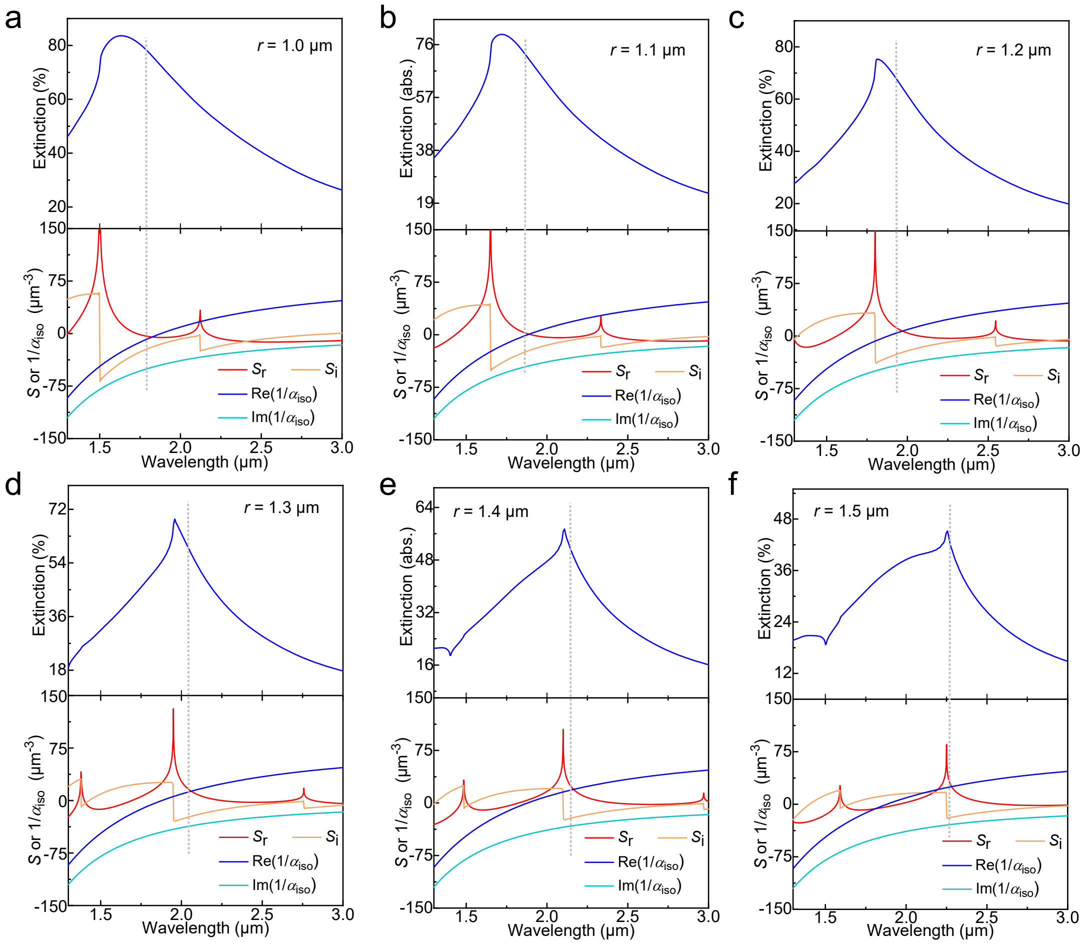

Supplementary Fig. 15 | Analysis of array factors and extinction spectra for square arrays with different periodic distances *r* = 1.0-1.5 μm, based on acid-treated PEDOT:ToS. The extinction spectra were obtained by FDTD simulations. The diameter of the nanodisks was set to 0.52 μm, and the height of the nanodisks was set to 0.3 μm.

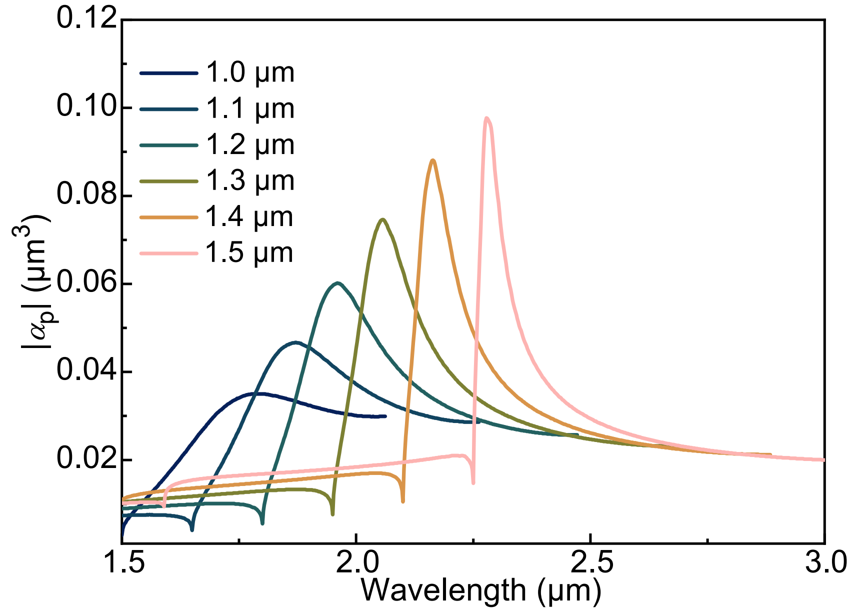

Supplementary Fig. 16 | Absolute periodic polarizability |α_p| of square-shaped arrays with different periodic distances r = 1.0-1.5 μm, based on acid-treated PEDOT:ToS nanoantennas with 0.52 μm diameter and 0.3 μm height.

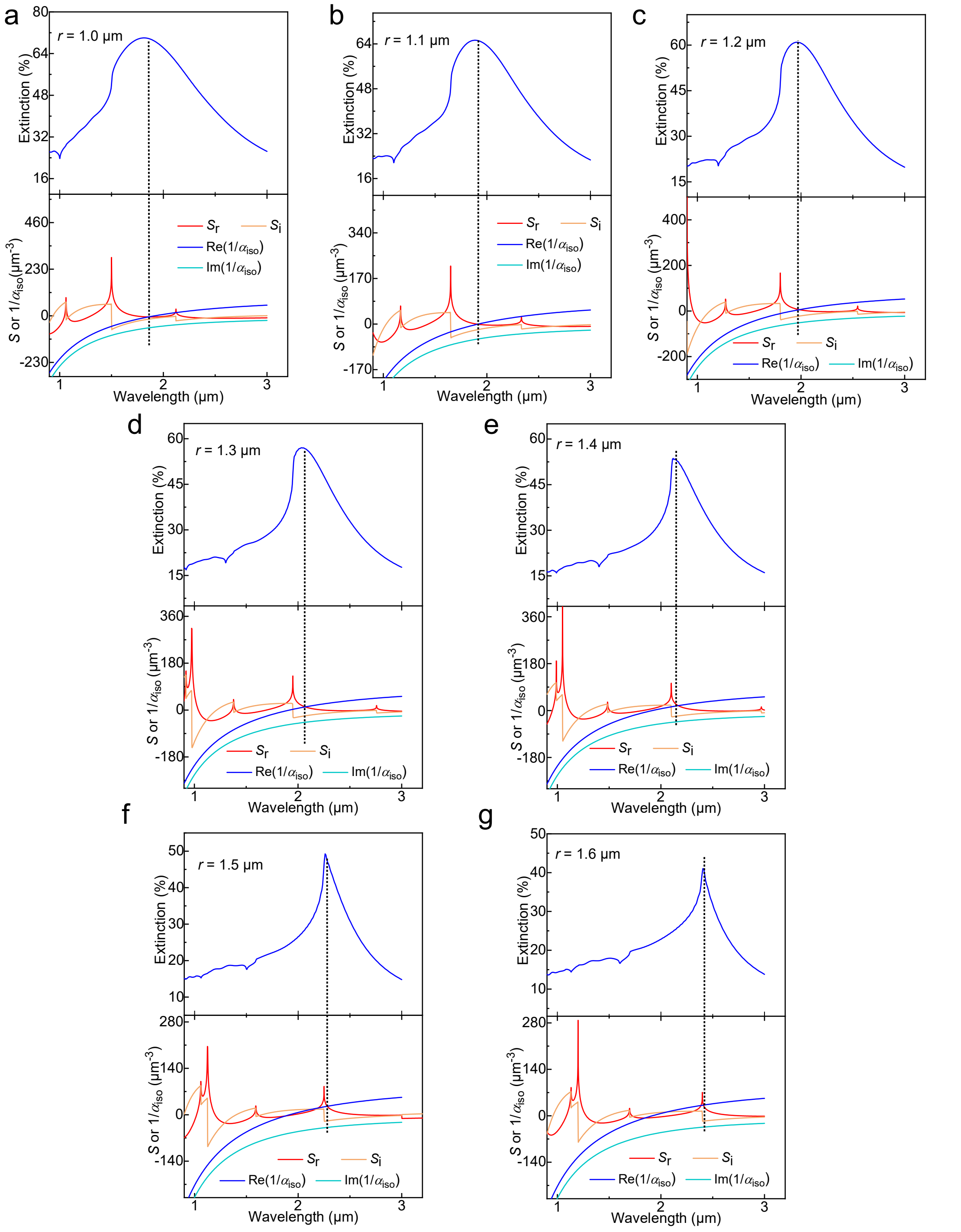

Supplementary Fig. 17 | Analysis of array factors and extinction spectra for square arrays with different periodic distances r = 1.0-1.6 μm, based on nanoantennas made from PEDOT:Sulf[1]. The extinction spectra were obtained by FDTD simulations. The diameter of nanodisks was set to 0.52 μm, and the height of nanodisks was set to 0.2 μm.

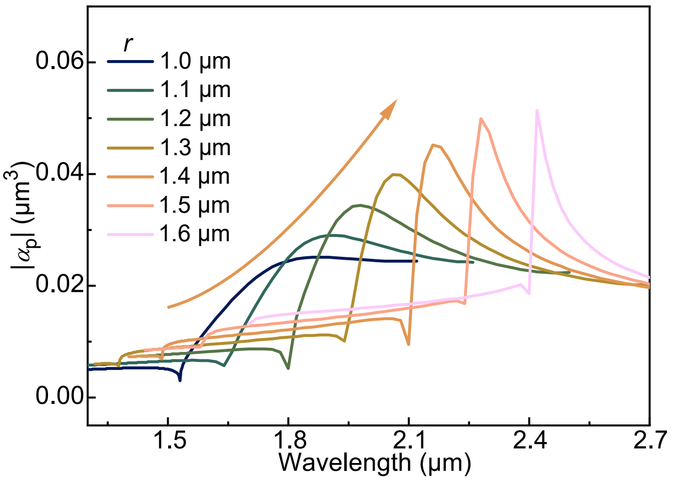

Supplementary Fig. 18 | Absolution values of periodic polarizability |α_p| in square-shaped array factors with different periodic distances r = 1.0-1.6 μm, based on PEDOT:Sulf[1]. The diameter of nanodisks was set to 0.52 μm, and the height of nanodisks was set to 0.2 μm. These peaks correspond to the surface plasmonic resonance based on the coupling interactions along the square-edges.

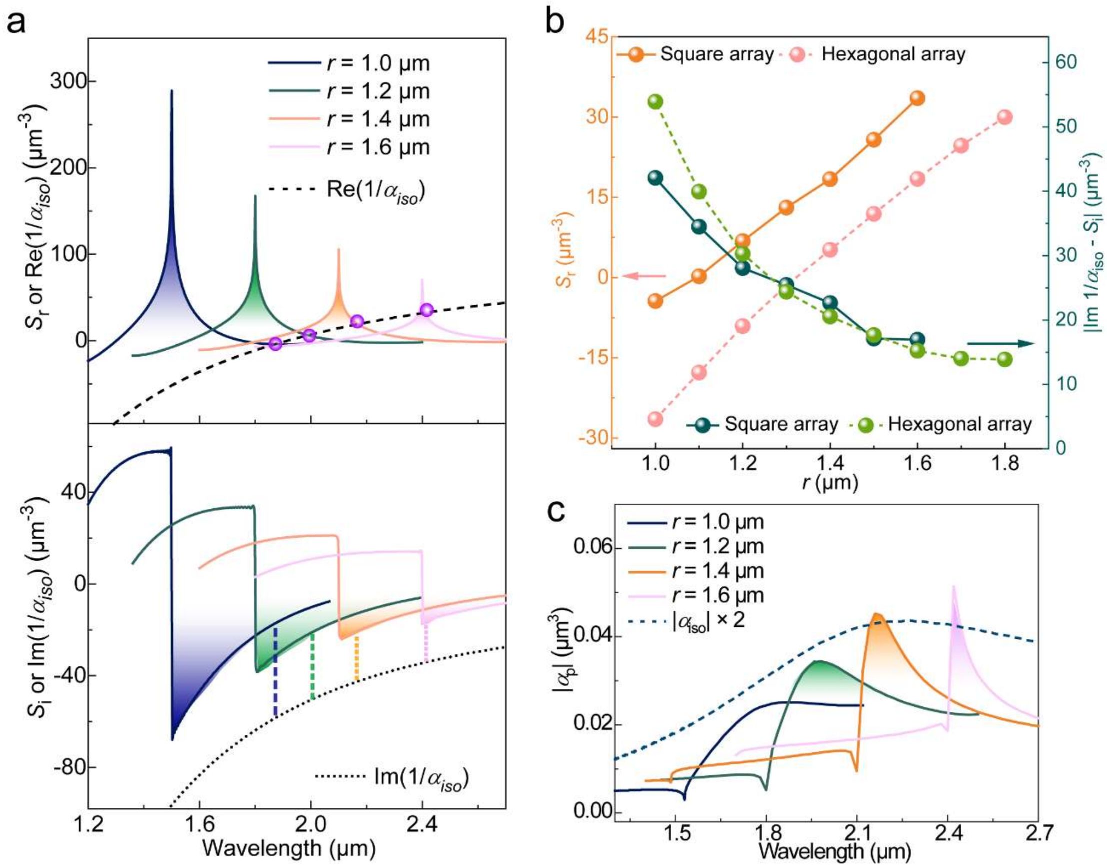

Supplementary Fig. 19 | Analysis of matching conditions of CLRs based on nanoantennas made from PEDOT:Sulf[1]. a Analysis of the real and imaginary part of array factors (S_r and S_i, respectively) with $1/\alpha_{iso}$, under the periodic distances r = 1.0, 1.2, 1.4, and, 1.6 μm. The diameter of nanodisks was set to 0.52 μm, and the height of nanodisks was set to 0.2 μm. **b** S_r and $|Im(1/\alpha_{iso})$ - $S_i|$ at λ_r, in both square and hexagonal arrays. **c** The magnitude of periodic polarizability ($|\alpha_p|$), along with the $|\alpha_{iso}|$ curve from LSPR.

Supplementary Note 2. Array factors combined with detuning wavelengths

The calculation of array factors (S) is based on Supplementary Equation (1), or Equation (1) in the manuscript)[2, 3]:

$$S = \sum_j^N \exp(i\mathbf{k}r_j)\left[\frac{(1-i\mathbf{k}r_j)(3\cos^2\theta_j - 1)}{r_j^3} + \frac{\mathbf{k}^2\sin^2\theta_j}{r_j}\right] \quad (2)$$

where r_j is the central distance between two nanoantennas, θ_j is the angle between the electrical field direction and the lattice vector direction, $\mathbf{k}$ is the wave vector ($\mathbf{k} = 2\pi n_s/\lambda$) with the wavelength λ, and, N is the number of other nanoantennas along the specific direction. In square-shaped arrays, considering the orientation of electrical polarizations decomposed into orthogonal directions (in a random manner), along with the coupling interactions in the diagonal direction of square arrays, the S values are calculated via the equation:

$$S = \sum_j^N \exp(i\mathbf{k}r_j)\left\{\left[\frac{(1-i\mathbf{k}r_j)[3\cos^2(\theta_j)-1]}{r_j^3} + \frac{\mathbf{k}^2\sin^2(\theta_j)}{r_j}\right] + \left[\frac{(1-i\mathbf{k}r_j)[3\cos^2(0.5\pi-\theta_j)-1]}{r_j^3} + \frac{\mathbf{k}^2\sin^2(0.5\pi-\theta_j)}{\sqrt{2}r_j}\right]\right\}$$

$$+\sum_j^N \exp(i\sqrt{2}\mathbf{k}r_j)\left\{\left[\frac{(1-i\sqrt{2}\mathbf{k}r_j)[3\cos^2(0.25\pi-\theta_j)-1]}{2\sqrt{2}r_j^3} + \frac{\mathbf{k}^2\sin^2(0.25\pi-\theta_j)}{\sqrt{2}r_j}\right] + \left[\frac{(1-i\sqrt{2}\mathbf{k}r_j)[3\cos^2(0.75\pi-\theta_j)-1]}{2\sqrt{2}r_j^3} + \frac{\mathbf{k}^2\sin^2(0.75\pi-\theta_j)}{\sqrt{2}r_j}\right]\right\}$$

(3)

As $\cos^2\theta_j+\cos^2(0.5\pi-\theta_j)=\sin^2\theta_j+\sin^2(0.5\pi-\theta_j)=1$ and $\cos^2(0.25\pi-\theta_j)+\cos^2(0.75\pi-\theta_j)=\sin^2(0.25\pi-\theta_j)+\sin^2(0.75\pi-\theta_j)=1$, the sin θ_j and cos θ_j parts are eliminated, which simplifies Supplementary Equation (3) to:

$$S = \sum_j^N \left\{\exp(i\mathbf{k}r_j)\left[\frac{(1-i\mathbf{k}r_j)}{r_j^3} + \frac{\mathbf{k}^2}{r_j}\right] + \exp(i\sqrt{2}\mathbf{k}r_j)\left[\frac{(1-i\mathbf{k}\sqrt{2}r_j)}{2\sqrt{2}r_j^3} + \frac{\mathbf{k}^2}{\sqrt{2}r_j}\right]\right\} \quad (4)$$

At the wavelength of surface plasmonic resonances ($\lambda = \lambda_r$), the wave vector should be:

$$|\mathbf{k}| = \frac{2\pi n_s}{\lambda_r} \quad (5)$$

Considering the calculation of normalized detuning wavelength[4] $\Delta = (\lambda_r - n_s r)/n_s r$, where r is the periodic distance, the wave vector can be transformed into Supplementary Equation (6):

$$|\mathbf{k}r| = \frac{2\pi n_s r}{\lambda_r} = \frac{2\pi}{1+\Delta} \quad (6)$$

Thus, the relationship between S and Δ is:

$$S(r,\Delta) = \frac{1}{(Nr)^3}\sum_1^N \left\{\left[1-\frac{i2N\pi}{1+\Delta}+\left(\frac{2N\pi}{1+\Delta}\right)^2\right]\exp\left(\frac{i2N\pi}{1+\Delta}\right) + \frac{1}{2\sqrt{2}}\left[1-\frac{i2\sqrt{2}N\pi}{1+\Delta}+\left(\frac{2\sqrt{2}N\pi}{\Delta+1}\right)^2\right]\exp\left(\frac{i2\sqrt{2}N\pi}{1+\Delta}\right)\right\} \quad (7)$$

According to the Euler equation, the real part of S (denoted as S_r) is:

$S_{\mathrm{r}}(r,\Delta) =$
$$\frac{1}{(Nr)^3}\sum_1^N \{ [1+\left(\frac{2N\pi}{1+\Delta}\right)^2] \cos\left(\frac{2N\pi}{1+\Delta}\right)+\frac{2N\pi}{1+\Delta}\sin\left(\frac{2N\pi}{1+\Delta}\right)+\frac{1}{2\sqrt{2}}[1+\left(\frac{2N\pi}{1+\Delta}\right)^2] \cos\left(\frac{2\sqrt{2}N\pi}{1+\Delta}\right)+\frac{1}{2\sqrt{2}}\frac{2N\pi}{1+\Delta}\sin\left(\frac{2\sqrt{2}N\pi}{1+\Delta}\right) \} \quad (8)$$

The imaginary part of S (denoted as S_{i}) is:

$$S_{\mathrm{i}}(r,\Delta)= \frac{1}{(Nr)^3}\sum_1^N \{ [1+\left(\frac{2N\pi}{1+\Delta}\right)^2] \sin\left(\frac{2N\pi}{1+\Delta}\right) - \frac{2N\pi}{1+\Delta}\cos\left(\frac{2N\pi}{1+\Delta}\right)+\frac{1}{2\sqrt{2}}\left[1+\left(\frac{2N\pi}{1+\Delta}\right)^2\right]\sin\left(\frac{2\sqrt{2}N\pi}{1+\Delta}\right) - \frac{1}{2\sqrt{2}}\frac{2N\pi}{1+\Delta}\cos\left(\frac{2\sqrt{2}N\pi}{1+\Delta}\right) \} \quad (9)$$

Supplementary Equation (8) and (9) can be further simplified if neglecting coupling interactions along the diagonal direction[5], and evaluating Δ approximate to 0:

$$S_{\mathrm{r}}(r,\Delta) = \frac{1}{(Nr)^3}\sum_1^N \left[1+\left(\frac{2N\pi}{1+\Delta}\right)^2 \right] \quad (10)$$

$$S_{\mathrm{i}}(r,\Delta) = \frac{1}{(Nr)^3}\sum_1^N \left(-\frac{2N\pi}{1+\Delta}\right) \quad (11)$$

According to Supplementary Equation (10) and (11), the decrease in Δ can make S_{r} more positive and S_{i} more negative, consistent with Fig. 2a and 2b in the manuscript, both of which show larger absolution values of array factors that indicates larger coupling interactions between adjacent nanoantennas.

Similarly, hexagonal arrays have an equation of array factor[6] according to:

$$S = \sum_j^N \exp(i\mathbf{k}r_{\mathrm{j}}) \left\{ \left[\frac{(1-i\mathbf{k}r_{\mathrm{j}})[3\cos^2\theta_{\mathrm{j}} -1]}{r_{\mathrm{j}}^3} + \frac{\mathbf{k}^2\sin^2\theta_{\mathrm{j}}}{r_{\mathrm{j}}}\right] + \left[\frac{(1-i\mathbf{k}r_{\mathrm{j}})[3\cos^2(\pi/3-\theta_{\mathrm{j}}) -1]}{r_{\mathrm{j}}^3} + \frac{\mathbf{k}^2\sin^2(\pi/3-\theta_{\mathrm{j}})}{r_{\mathrm{j}}}\right] + \left[\frac{(1-i\mathbf{k}r_{\mathrm{j}})[3\cos^2(2\pi/3-\theta_{\mathrm{j}}) -1]}{r_{\mathrm{j}}^3} + \frac{\mathbf{k}^2\sin^2(2\pi/3-\theta_{\mathrm{j}})}{r_{\mathrm{j}}}\right] \right\} +$$
$$\sum_j^N \exp(i\sqrt{3}\mathbf{k}r_{\mathrm{j}}) \left\{ \left[\frac{(1-i\sqrt{3}\mathbf{k}r_{\mathrm{j}})[3\cos^2(\pi/6-\theta_{\mathrm{j}}) -1]}{3\sqrt{3}r_{\mathrm{j}}^3} + \frac{\mathbf{k}^2\sin^2(\pi/6-\theta_{\mathrm{j}})}{\sqrt{3}r_{\mathrm{j}}}\right] + \left[\frac{(1-i\sqrt{3}\mathbf{k}r_{\mathrm{j}})[3\cos^2(\pi/2-\theta_{\mathrm{j}}) -1]}{3\sqrt{3}r_{\mathrm{j}}^3} + \frac{\mathbf{k}^2\sin^2(\pi/2-\theta_{\mathrm{j}})}{\sqrt{3}r_{\mathrm{j}}}\right] + \left[\frac{(1-i\sqrt{3}\mathbf{k}r_{\mathrm{j}})[3\cos^2(5\pi/6-\theta_{\mathrm{j}}) -1]}{3\sqrt{3}r_{\mathrm{j}}^3} + \frac{\mathbf{k}^2\sin^2(5\pi/6-\theta_{\mathrm{j}})}{\sqrt{3}r_{\mathrm{j}}}\right] \right\} \quad (12)$$

The sin θ_{j} and cos θ_{j} parts are also eliminated, because $\cos^2\theta_{\mathrm{j}}+\cos^2(\pi/3-\theta_{\mathrm{j}})+\cos^2(2\pi/3-\theta_{\mathrm{j}})=\cos^2\theta_{\mathrm{j}}+(\frac{\sqrt{3}}{2}\sin\theta_{\mathrm{j}}+\frac{1}{2}\cos\theta_{\mathrm{j}})^2+(\frac{\sqrt{3}}{2}\sin\theta_{\mathrm{j}}-\frac{1}{2}\cos\theta_{\mathrm{j}})^2=\frac{3}{2}$ and $\cos^2(\pi/6-\theta_{\mathrm{j}})+\cos^2(\pi/2-\theta_{\mathrm{j}})+\cos^2(5\pi/6-\theta_{\mathrm{j}})=\sin^2\theta_{\mathrm{j}}+(\frac{1}{2}\sin\theta_{\mathrm{j}}+\frac{\sqrt{3}}{2}\cos\theta_{\mathrm{j}})^2+(\frac{1}{2}\sin\theta_{\mathrm{j}}-\frac{\sqrt{3}}{2}\cos\theta_{\mathrm{j}})^2=\frac{3}{2}$. Thus, Supplementary Equation (12) can be simplified to:

$$S = \frac{3}{2}\left[\sum_j^N \exp(i\mathbf{k}r_{\mathrm{j}})\left(\frac{1-i\mathbf{k}r_{\mathrm{j}}}{r_{\mathrm{j}}^3}+\frac{\mathbf{k}^2}{r_{\mathrm{j}}}\right) + \sum_j^N \exp(i\sqrt{3}\mathbf{k}r_{\mathrm{j}})\left(\frac{1-i\sqrt{3}\mathbf{k}r_{\mathrm{j}}}{3\sqrt{3}r_{\mathrm{j}}^3}+\frac{\mathbf{k}^2}{\sqrt{3}r_{\mathrm{j}}}\right)\right] \quad (13)$$

At the resonance wavelength $\lambda = \lambda_{\mathrm{r}}$, S can be combined with Δ:

$$S(r,\Delta)=\frac{3}{2}\frac{1}{(Nr)^3}\sum_1^N\{[1-\frac{i2N\pi}{1+\Delta}+\left(\frac{2N\pi}{1+\Delta}\right)^2]\exp(\frac{i2N\pi}{1+\Delta})+\frac{1}{3\sqrt{3}}\left[1-\frac{i3\sqrt{3}N\pi}{1+\Delta}+\left(\frac{3\sqrt{3}N\pi}{\Delta+1}\right)^2\right]\exp(\frac{i3\sqrt{3}N\pi}{1+\Delta})\} \quad (14)$$

or when shifting the constant "3/2" to *Nr* part

$$S(r,\Delta)=\frac{1}{(N\sqrt[3]{\frac{2}{3}}r)^3}\sum_1^N\{[1-\frac{i2N\pi}{1+\Delta}+\left(\frac{2N\pi}{1+\Delta}\right)^2]\exp(\frac{i2N\pi}{1+\Delta})+\frac{1}{3\sqrt{3}}\left[1-\frac{i3\sqrt{3}N\pi}{1+\Delta}+\left(\frac{3\sqrt{3}N\pi}{\Delta+1}\right)^2\right]\exp(\frac{i3\sqrt{3}N\pi}{1+\Delta})\} \quad (15)$$

In this case, the resonance wavelength λ_r from hexagonal arrays should obey the law (if Δ is approximate to 0):

$$\lambda_r=\sqrt[3]{\frac{2}{3}}n_s r \quad (16)$$

The simplified S_r and S_i for hexagonal arrays are:

$$S_r(r,\Delta)=\frac{3}{2}\frac{1}{(Nr)^3}\sum_1^N[1+\left(\frac{2N\pi}{1+\Delta}\right)^2] \quad (17)$$

$$S_i(r,\Delta)=\frac{3}{2}\frac{1}{(Nr)^3}\sum_1^N(-\frac{2N\pi}{1+\Delta}) \quad (18)$$

Similar to those in square-shaped arrays, reducing Δ can also increase $|S_r|$ and $|S_i|$ values. However, under the same periodic distance, array factors in hexagonal arrays are around 1.5 times as large as those in square arrays, consistent with stronger coupling interactions[6] from 6 adjacent nanoantennas than coupling interactions from 4 neighboring nanoantennas in square-shaped arrays.

Supplementary Note 3. CLR in hexagonal arrays

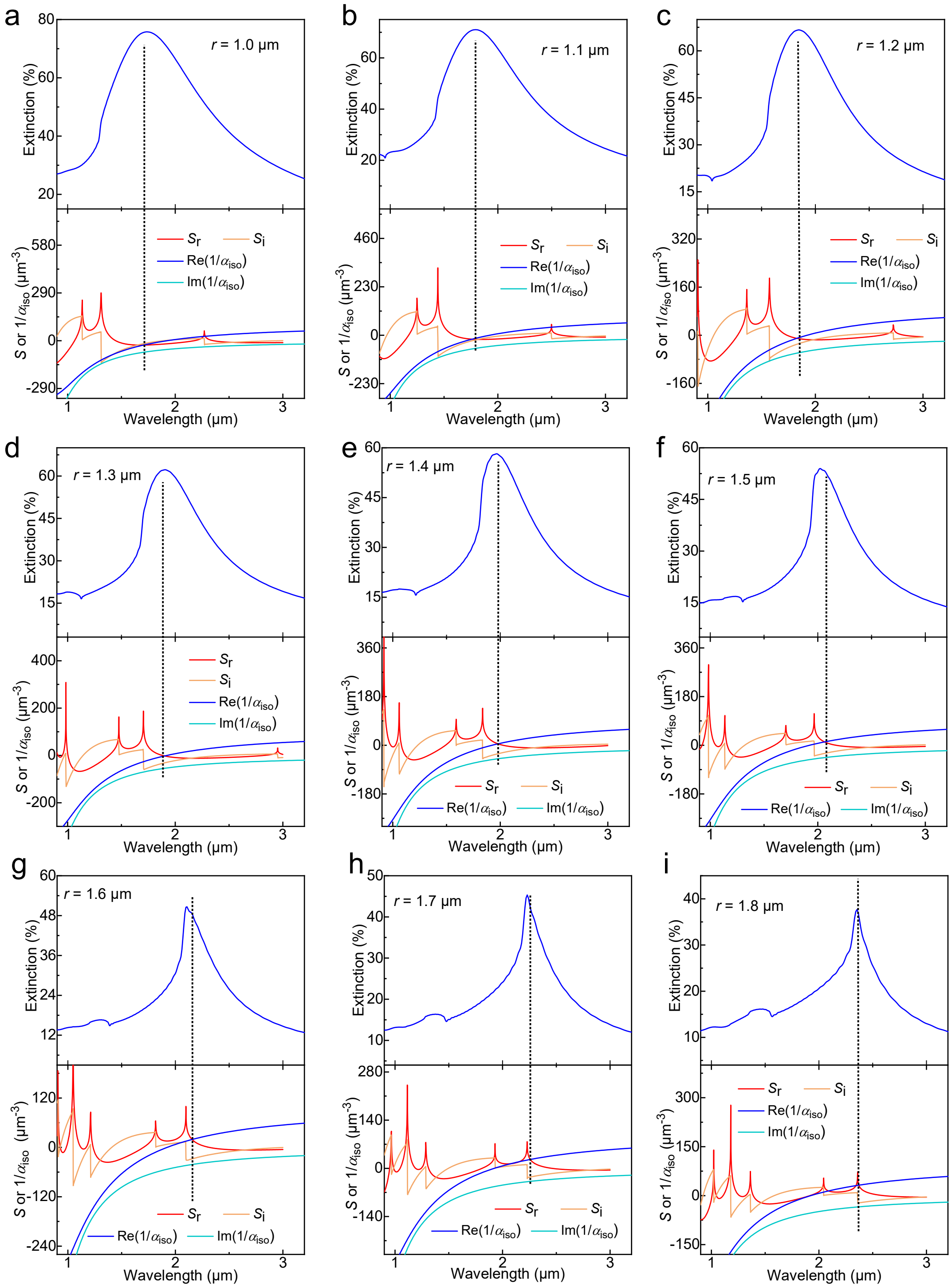

Supplementary Fig. 20 | Analysis of factors and extinction spectra in hexagonal arrays with different periodic distances r = 1.0-1.8 μm, based on acid-treated PEDOT:ToS. The extinction

spectra are based on the FDTD simulations. The diameter of nanodisks was set to 0.52 μm, and the height of nanodisks was set to 0.2 μm.

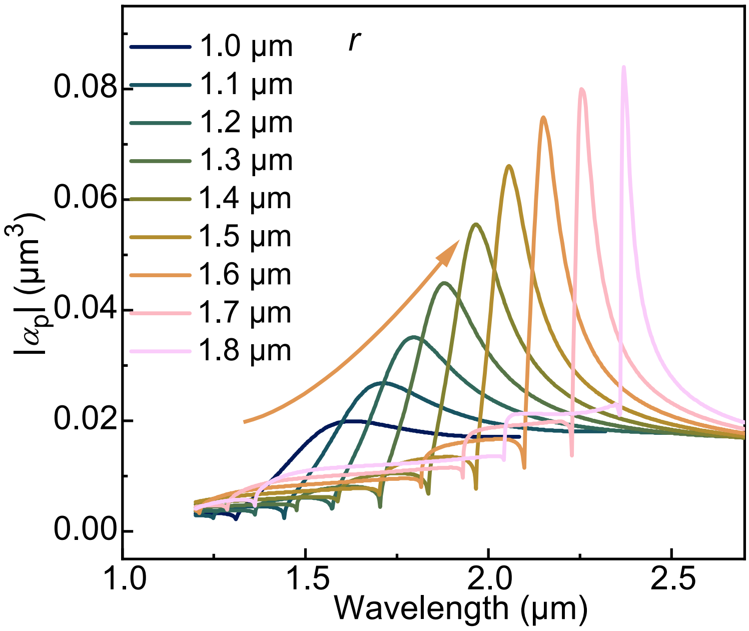

Supplementary Fig. 21 | Absolute periodic polarizability $|\alpha_p|$ for hexagonal array with different periodic distances *r* = 1.0-1.8 μm, based on acid-treated PEDOT:ToS. The diameter of nanodisks was set to 0.52 μm, and the height of nanodisks was set to 0.2 μm. These dominant peaks correspond to the surface plasmonic resonance based on the coupling interactions along the hexagonal-edges.

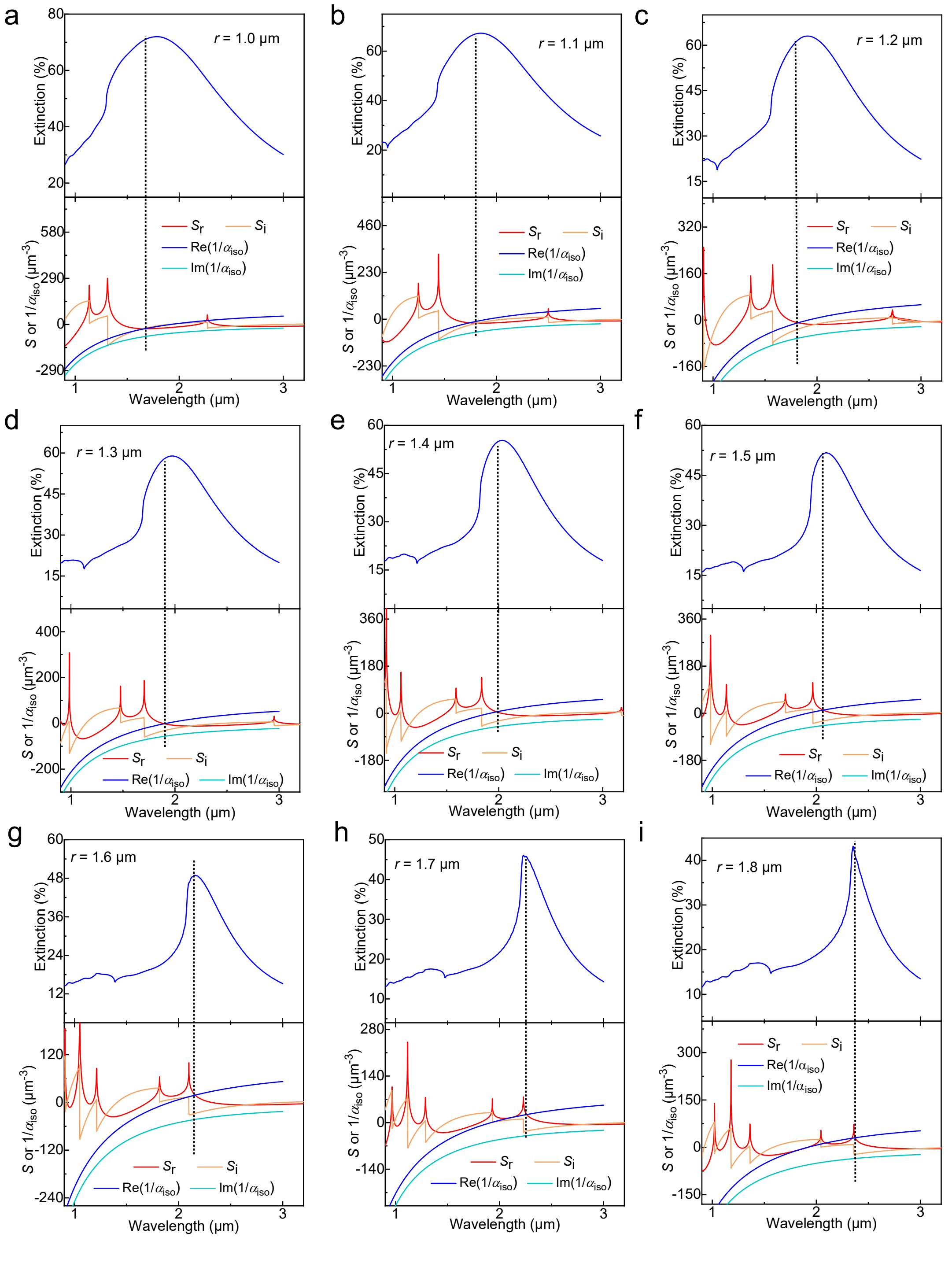

Supplementary Fig. 22 | Analysis of factors and extinction spectra in hexagonal arrays with different periodic distances *r* = 1.0-1.8 μm, based on PEDOT:Sulf[1]. The extinction spectra are based on the FDTD simulations. The diameter of nanodisks was set to 0.52 μm, and the height of nanodisks was set to 0.2 μm.

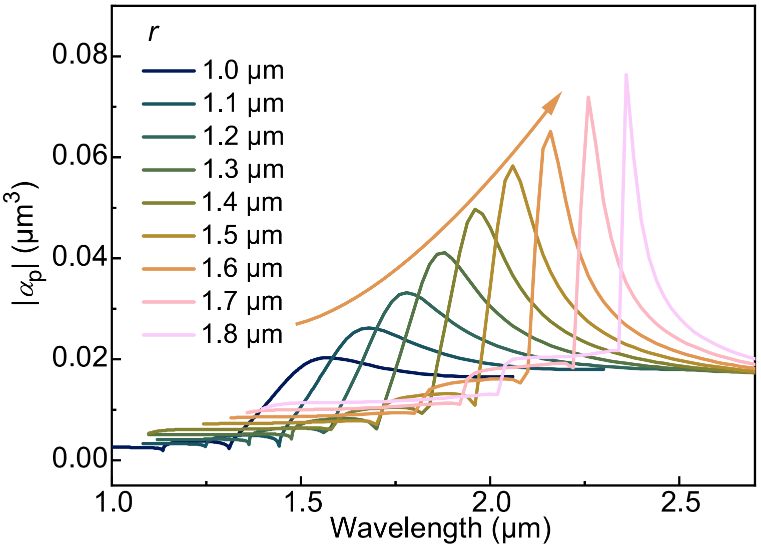

Supplementary Fig. 23 | Absolution values of periodic polarizability $|\alpha_p|$ in hexagonal array factors with different periodic distances r = 1.0-1.8 μm, based on PEDOT:Sulf[1]. The diameter of nanodisks was set to 0.52 μm, and the height of nanodisks was set to 0.2 μm. These dominant peaks correspond to the surface plasmonic resonance based on the coupling interactions along the hexagonal-edges.

Supplementary Note 4. Experimental results of PEDOT-based periodic arrays

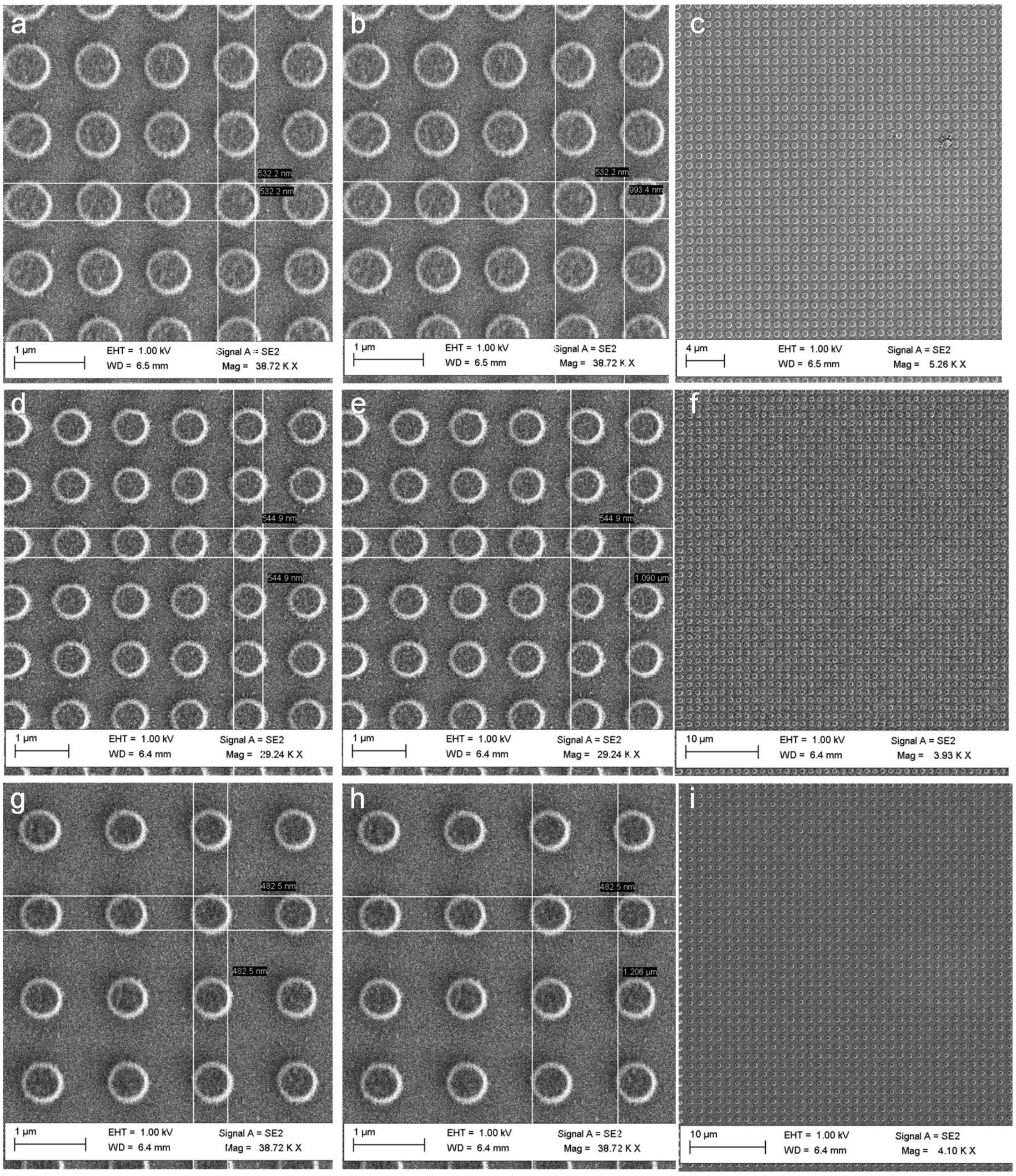

Supplementary Fig. 24 | SEM images of PEDOT-based square arrays (*r* = 1.0~1.2 μm) made through electron beam lithography. **a-c** for *r* = 1.0 μm; **d-f** for *r* = 1.1 μm; **g-i** for *r* = 1.2 μm. The substrate was glass slides and the nanoantenna material was acid-treated PEDOT:ToS.

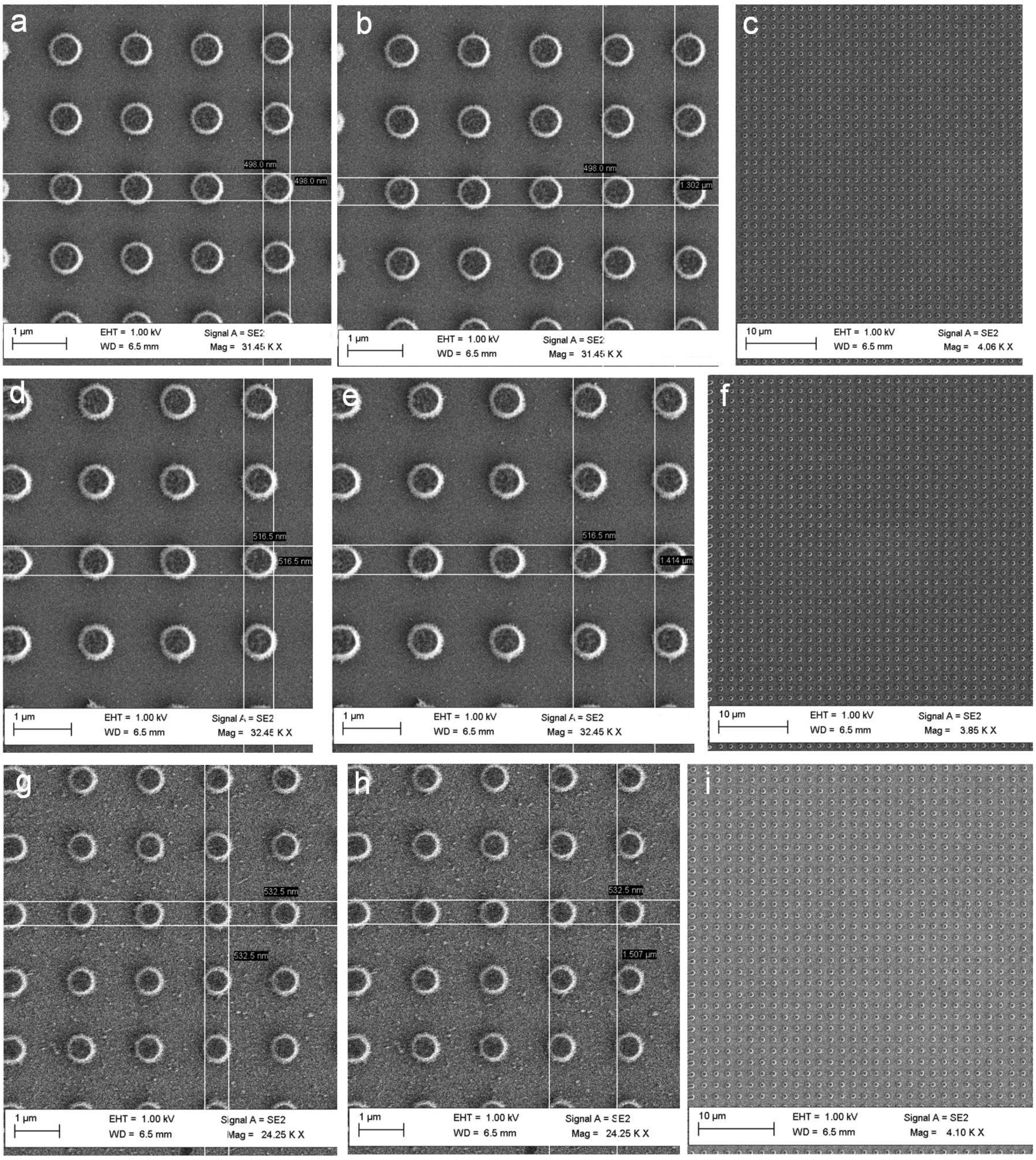

Supplementary Fig. 25 | SEM images of PEDOT-based square arrays (*r* = 1.3~1.5 µm) made through electron beam lithography. **a-c** for *r* = 1.3 µm; **d-f** for *r* = 1.4 µm; **g-i** for *r* = 1.5 µm. The substrate was glass slides and the nanoantenna material was acid-treated PEDOT:ToS.

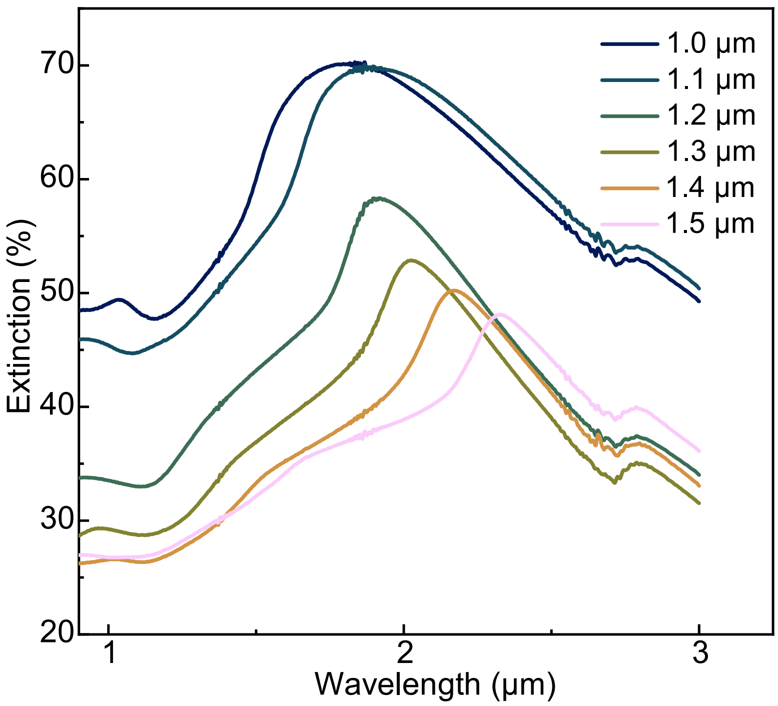

Supplementary Fig. 26 | Absolute extinction spectra (experimental results) of PEDOT-based periodic arrays (square shape) with various periodic distances *r* = 1.0~1.5 μm. The nanoantennas were made from acid-treated PEDOT:ToS (the diameters were 0.48-0.54 μm, and the height was 0.2 μm) on glass.

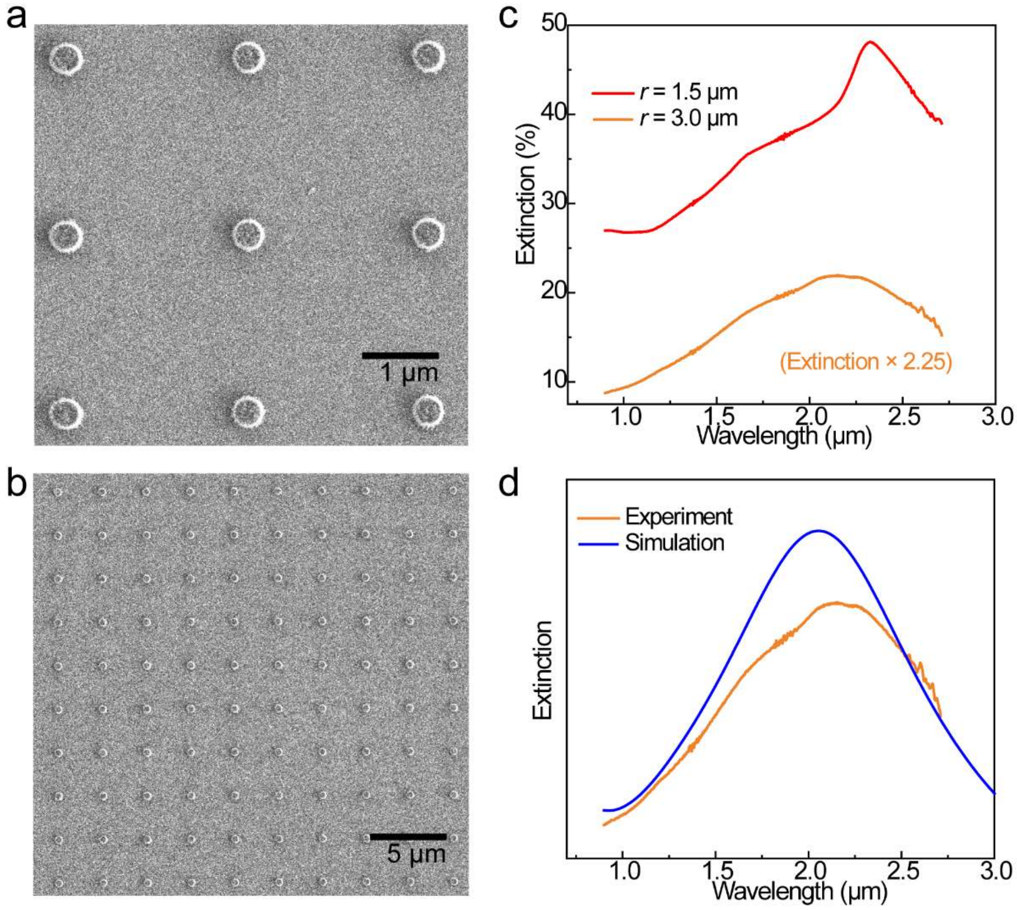

Supplementary Fig. 27 | Absolute extinction spectra of PEDOT-based periodic arrays (square shape) with larger periodic distances *r* = 3.0 μm. a-b SEM images of nanoantenna arrays (for nanoantenna units: the diameter of 0.51 μm, the height of 0.2 μm). **c** Experimental extinction spectra of PEDOT-based periodic arrays with $r = 3.0$ μm (marked in orange line) and $r = 1.5$ μm. Considering the number of nanoantenna units for $r = 1.5$ μm is 2.25 times more than those for $r = 3.0$ μm, we multiply 2.25 with extinction intensity for $r = 3.0$ μm. **d** Comparison with the FDTD simulations of the localized models (without periodic boundaries, marked in blue line). The nanoantennas were made from acid-treated PEDOT:ToS on glass.

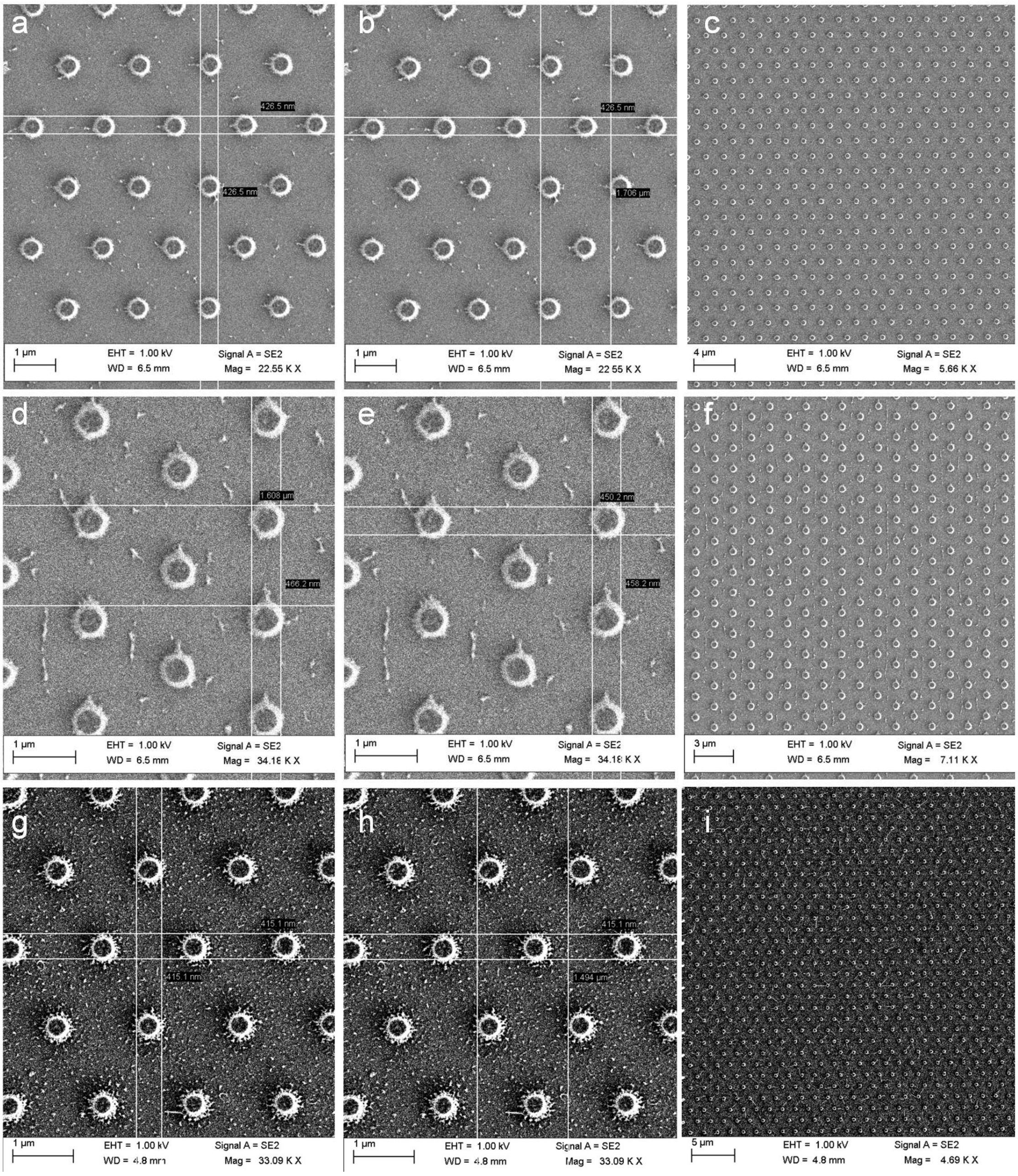

Supplementary Fig. 28 | SEM images of PEDOT-based hexagonal arrays (*r* = 1.5~1.7 µm) made through electron beam lithography. a-c for *r* = 1.7 µm; **d-f** for *r* = 1.6 µm; **g-i** for *r* = 1.5 µm. The substrate was glass slides and the nanoantenna material was acid-treated PEDOT:ToS. The diameters were 0.48-0.54 µm, and the height was 0.2 µm.

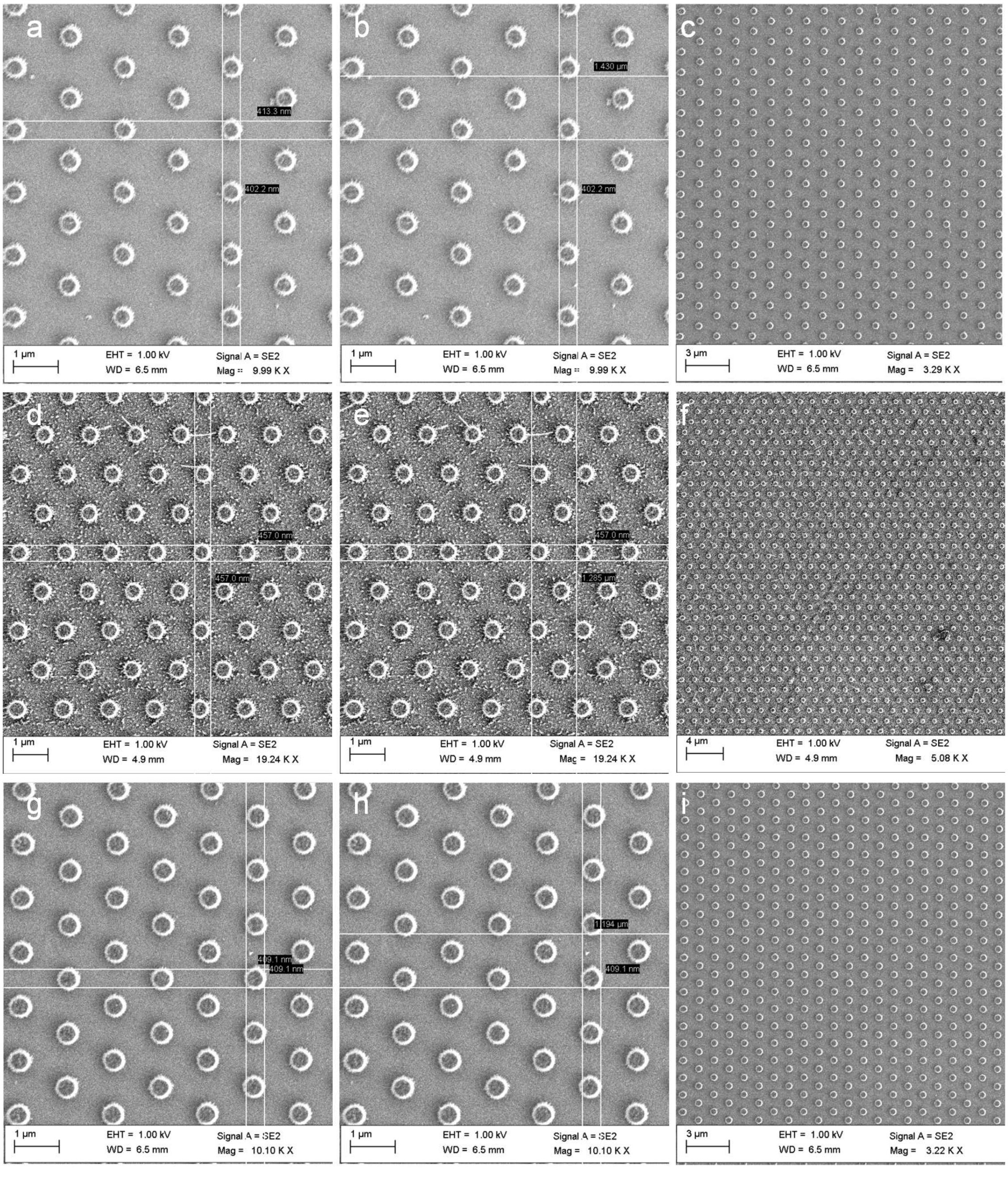

Supplementary Fig. 29 | SEM images of PEDOT-based hexagonal arrays (*r* = 1.2~1.4 μm) made through electron beam lithography. a-c for *r* = 1.4 μm; **d-f** for *r* = 1.3 μm; **g-i** for *r* = 1.2 μm. The substrate was glass slides and the nanoantenna material was acid-treated PEDOT:ToS. The diameters were 0.4-0.5 μm, and the height was 0.2 μm.

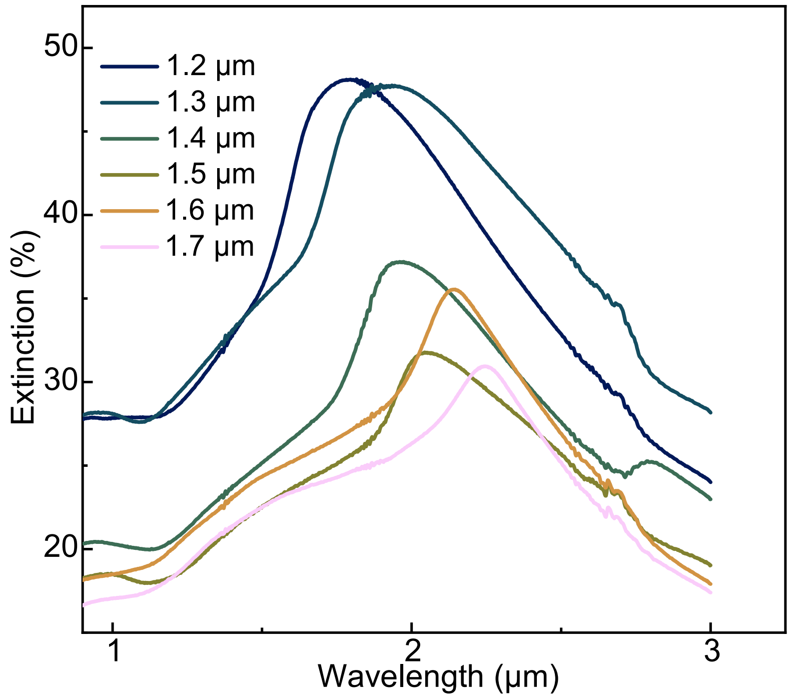

Supplementary Fig. 30 | Absolute extinction spectra (experimental results) of PEDOT-based periodic arrays (hexagonal shape) with various periodic distances *r* = 1.2~1.7 μm. The nanoantennas were made from acid-treated PEDOT:ToS (the diameters were 0.4-0.5 μm, and the height was 0.2 μm) on glass.

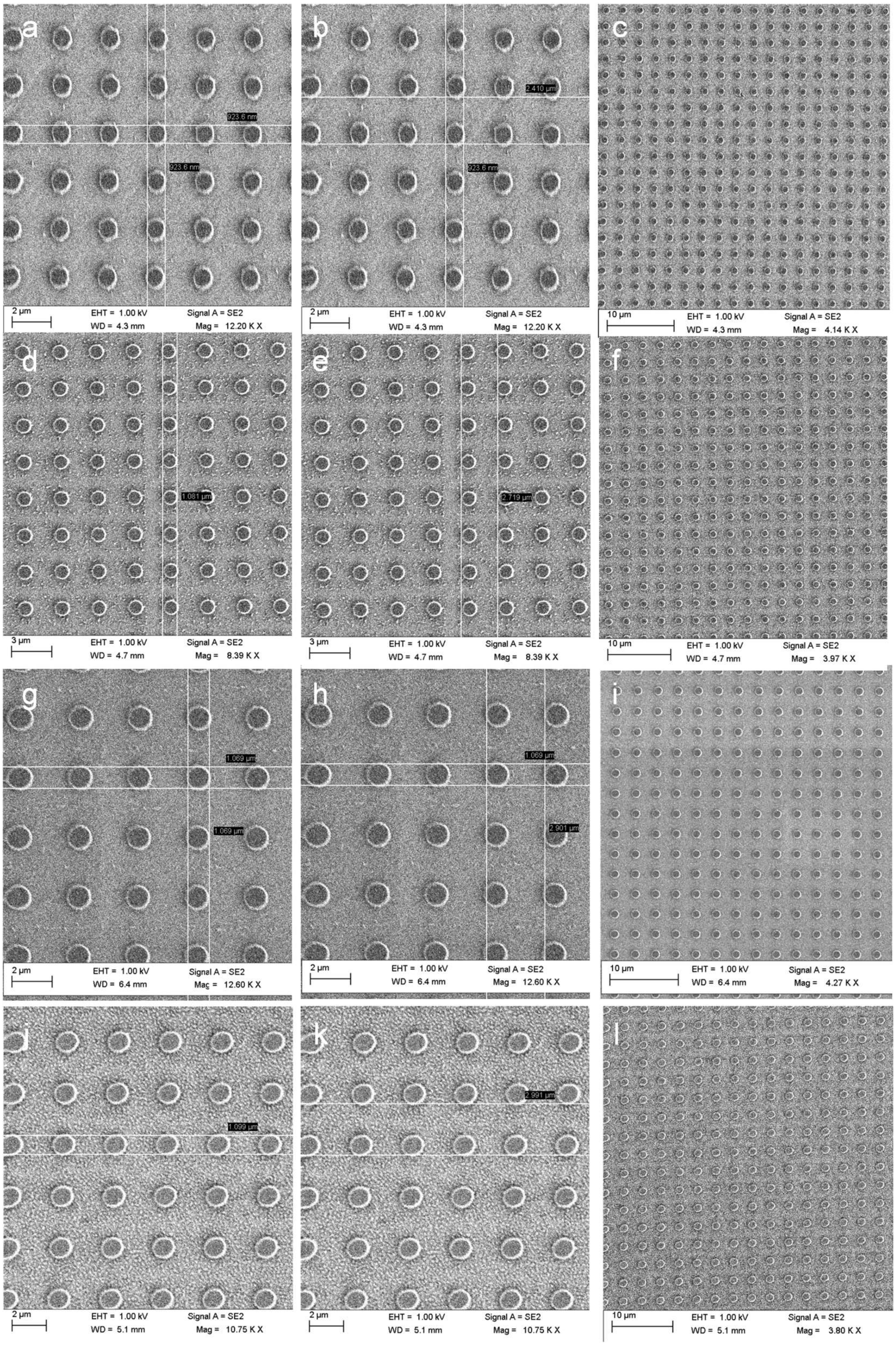

Supplementary Fig. 31 | SEM images of PEDOT-based square arrays (*r* = 2.4~3 µm) made through electron beam lithography. **a-c** for *r* = 2.4 µm; **d-f** for *r* = 2.7 µm; **g-i** for *r* = 2.9 µm; **j-l** for

r = 3.0 μm. The substrate was CaF_2 slides and the nanoantenna material was acid-treated PEDOT:ToS. The diameters were 0.9-1.1 μm, and the height was 0.2 μm.

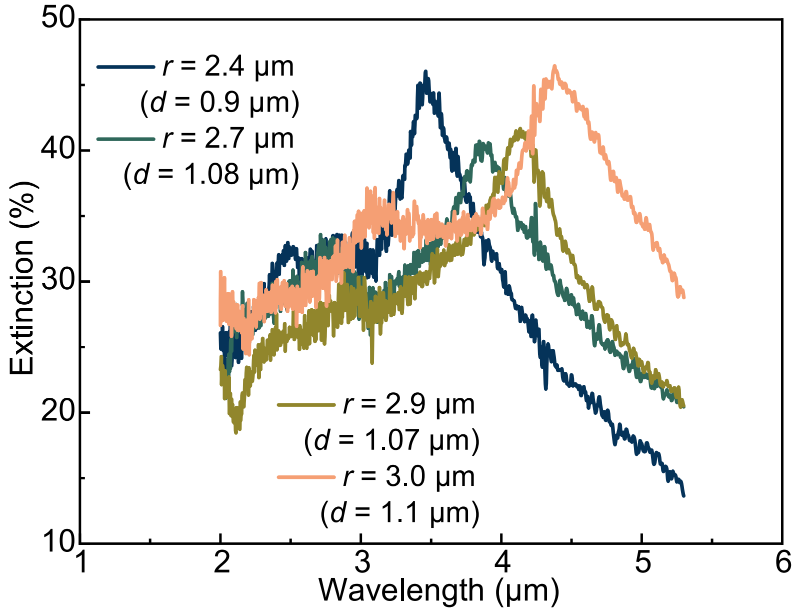

Supplementary Fig. 32 | Absolute extinction spectra (experimental results) of PEDOT-based periodic arrays (square shape) with various periodic distances r = 2.4~3.0 μm. The nanoantennas were made from acid-treated PEDOT:ToS (the diameters were 0.9-1.1 μm, and the height was 0.2 μm) on CaF_2 slides.

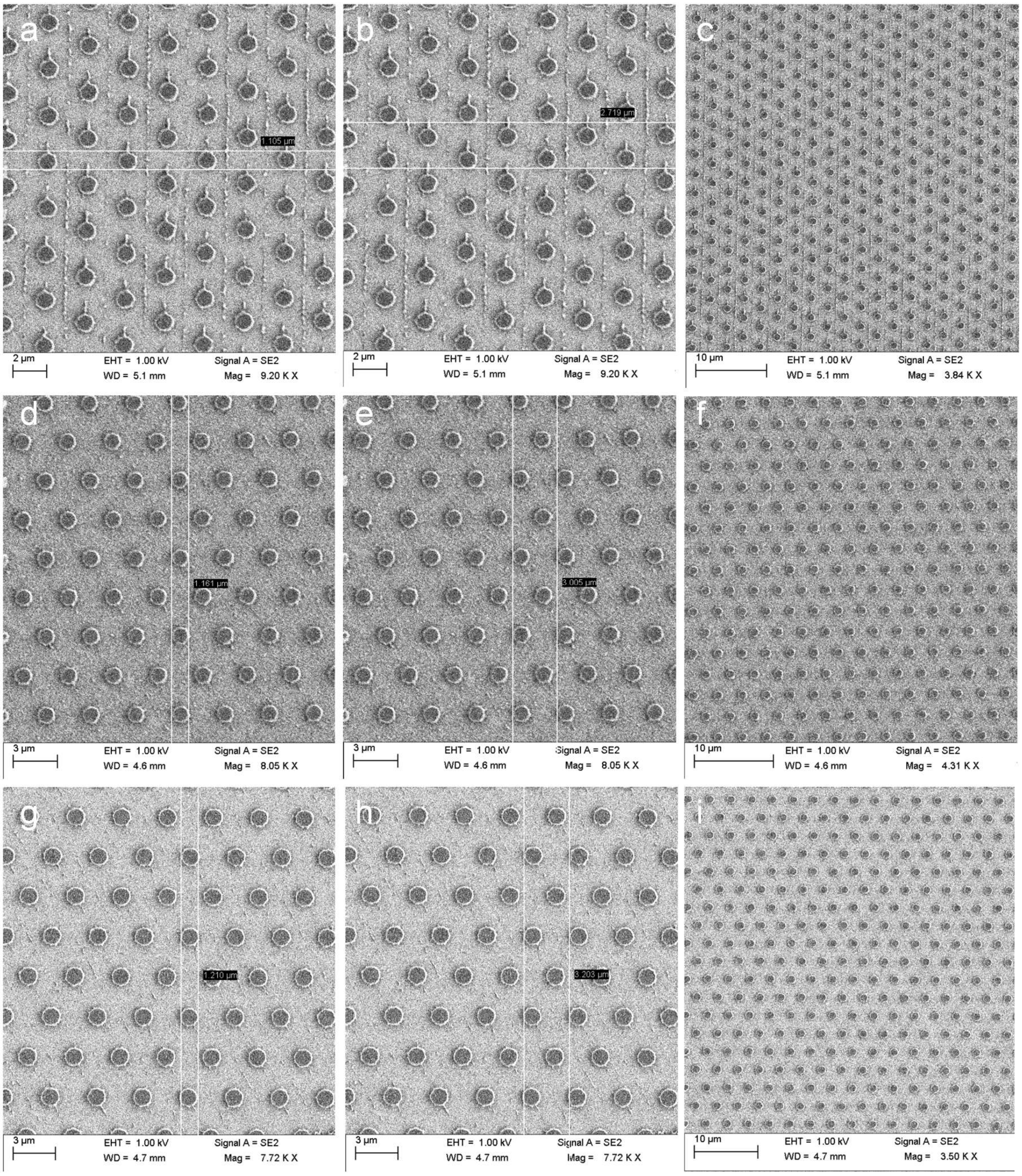

Supplementary Fig. 33 | SEM images of PEDOT-based hexagonal arrays (*r* = 2.7~3.2 µm) made through electron beam lithography. a-c for *r* = 2.7 µm; **d-f** for *r* = 3.0 µm; **g-i** for *r* = 3.2 µm. The substrate was CaF_2 slides and the nanoantenna material was acid-treated PEDOT:ToS. The diameters were 1.1-1.2 µm, and the height was 0.2 µm.

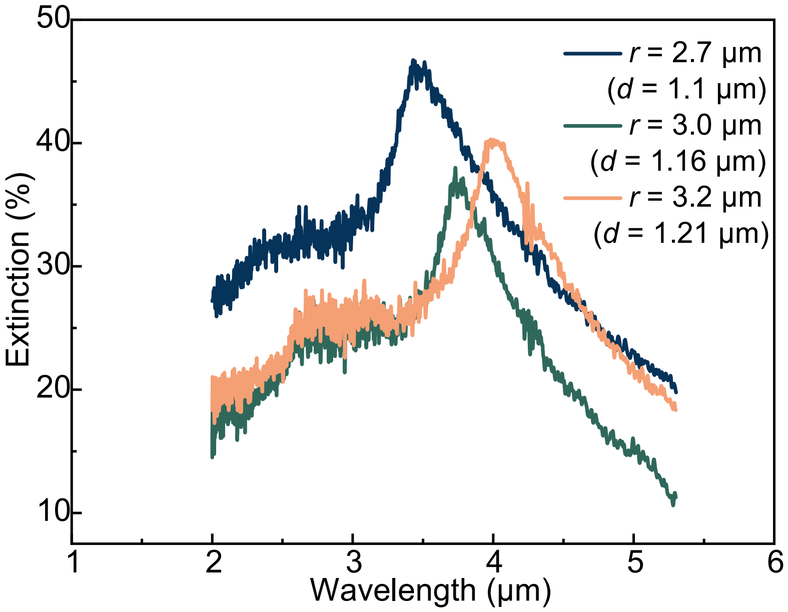

Supplementary Fig. 34 | Absolute extinction spectra (experimental results) of PEDOT-based periodic arrays (hexagonal shape) with various periodic distances *r* = 2.7~3.0 μm. The nanoantennas were made from acid-treated PEDOT:ToS (the diameters were 1.1-1.2 μm, and the height was 0.2 μm) on CaF_2 slides.

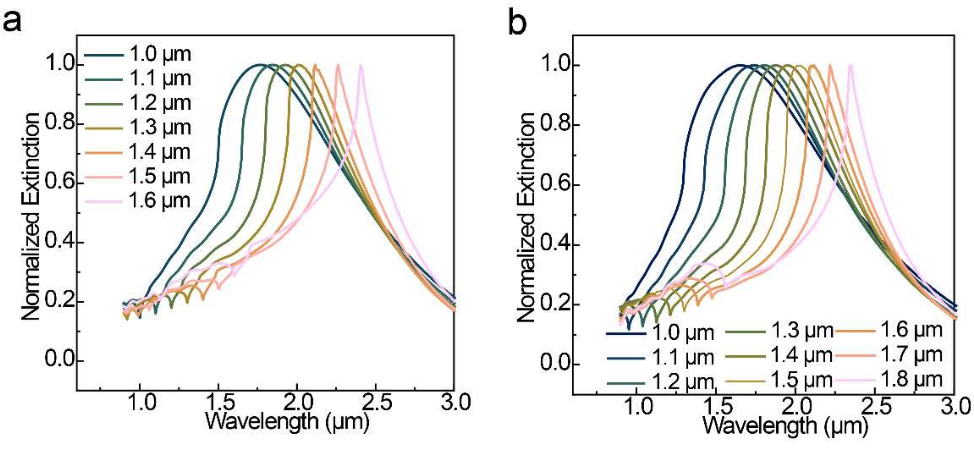

Supplementary Fig. 35 | FDTD simulations of *r*-dependent extinction spectra based on PEDOT:Sulf[1]. a in square arrays; **b** in hexagonal arrays. The diameter of nanodisks was set to 0.52 μm, and the height of nanodisks was set to 0.2 μm.

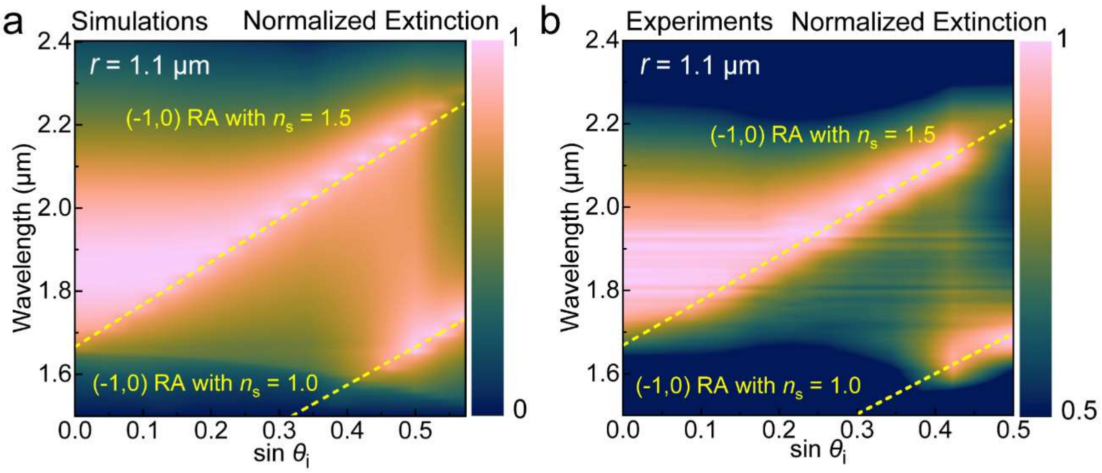

Supplementary Fig. 36 | Simulation (a) and experimental results (b) of angle-dependent extinction spectra for a square array with r = 1.1 μm. For FDTD simulations, the diameter of nanodisks was set to 0.52 μm, and the height of nanodisks was set to 0.2 μm. For experimental results, the diameter of nanodisks was 0.54 μm, and the height of nanodisks was 0.2 μm.

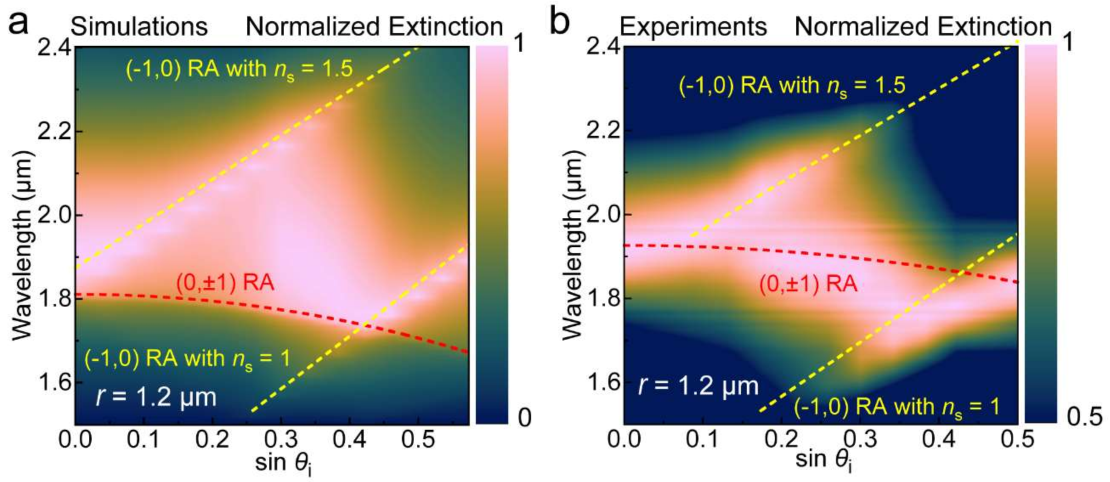

Supplementary Fig. 37 | Simulation (a) and experimental results (b) of the angle-dependent extinction spectra under the periodic distance r = 1.2 μm. For FDTD simulations, the diameter of nanodisks was set to 0.52 μm, and the height of nanodisks was set to 0.2 μm. For experimental results, the diameter of nanodisks was 0.48 μm, and the height of nanodisks was 0.2 μm.

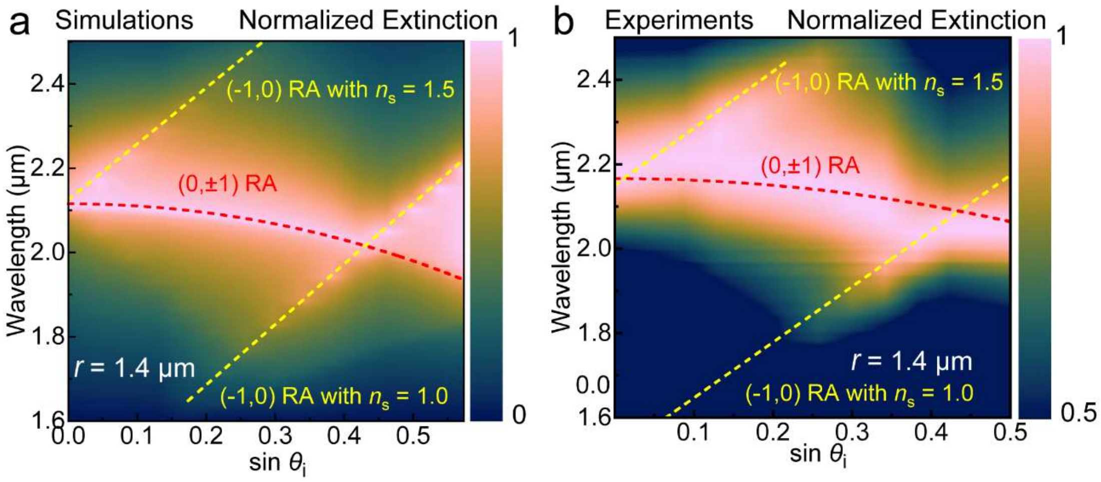

Supplementary Fig. 38 | Simulation (a) and experimental results (b) of the angle-dependent extinction spectra under the periodic distance *r* = 1.4 μm. For FDTD simulations, the diameter of nanodisks was set to 0.52 μm, and the height of nanodisks was set to 0.2 μm. For experimental results, the diameter of nanodisks was 0.51 μm, and the height of nanodisks was 0.2 μm.

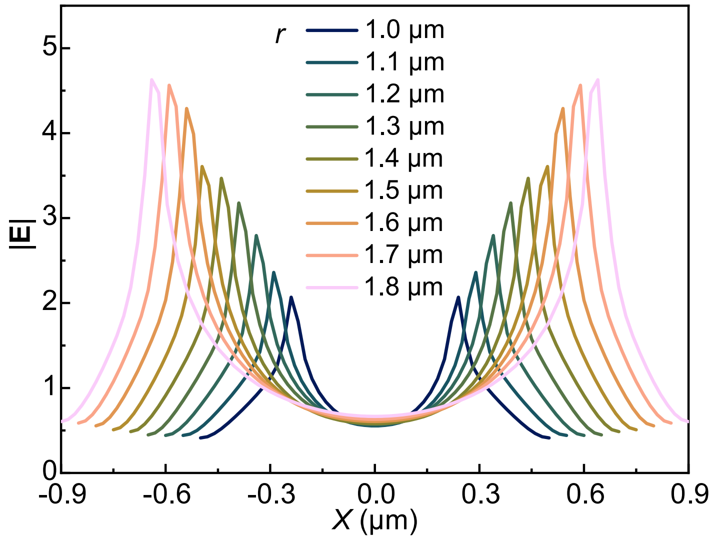

Supplementary Fig. 39 | Electrical field distribution (denoted by the magnitude |E|) for hexagonal arrays with different *r* = 1.0-1.8 μm at λ_r. In these hexagonal arrays, four PEDOT nanoantenna centers locate at the coordinate ($X = 0$, $Y = \sqrt{3}r/2$), ($X = 0$, $Y = -\sqrt{3}r/2$), ($X = r/2$, $Y = 0$), and ($X = -r/2$, $Y = 0$). At $Y = 0$, two nanoantenna centers located at $X = \pm r/2$ for respective periodicity. The diameter of PEDOT nanoantennas is set as $d = 0.52$ μm, and thus the maximum magnitude of electrical field (at hot spot regions) is distributed around $X = r/2 - d/2$ and $X = -r/2 + d/2$. The Z coordinate for electrical field distribution is set at the interface between PEDOT nanoantennas and the dielectric substrate.

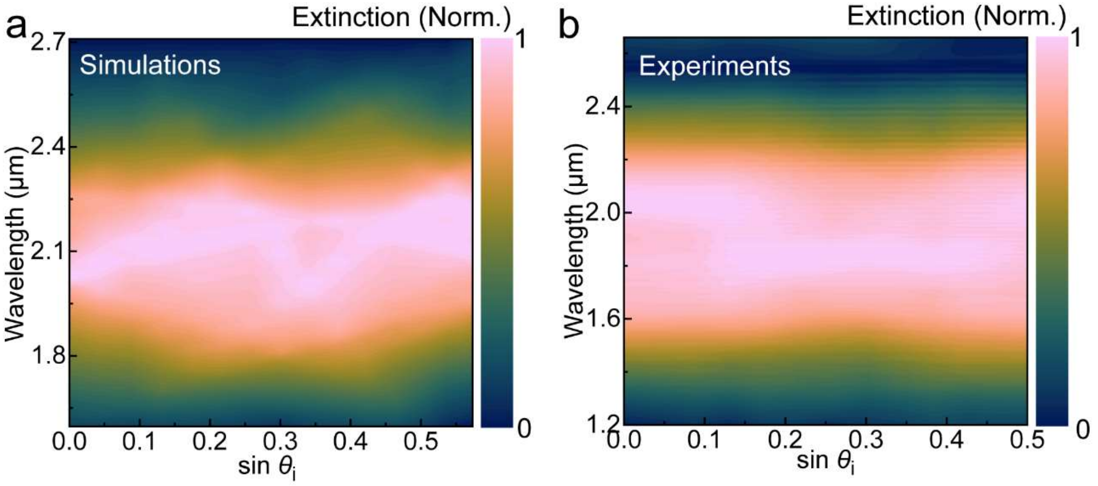

Supplementary Fig. 40 | Simulation (a) and experimental results (b) of the angle-dependent extinction spectra for the periodic distance *r* = 3.0 μm. For FDTD simulations, the diameter of nanodisks was set to 0.52 μm, and the height of nanodisks was set to 0.2 μm. For experimental results, the diameter of nanodisks was ~0.51 μm, and the height of nanodisks was 0.2 μm.

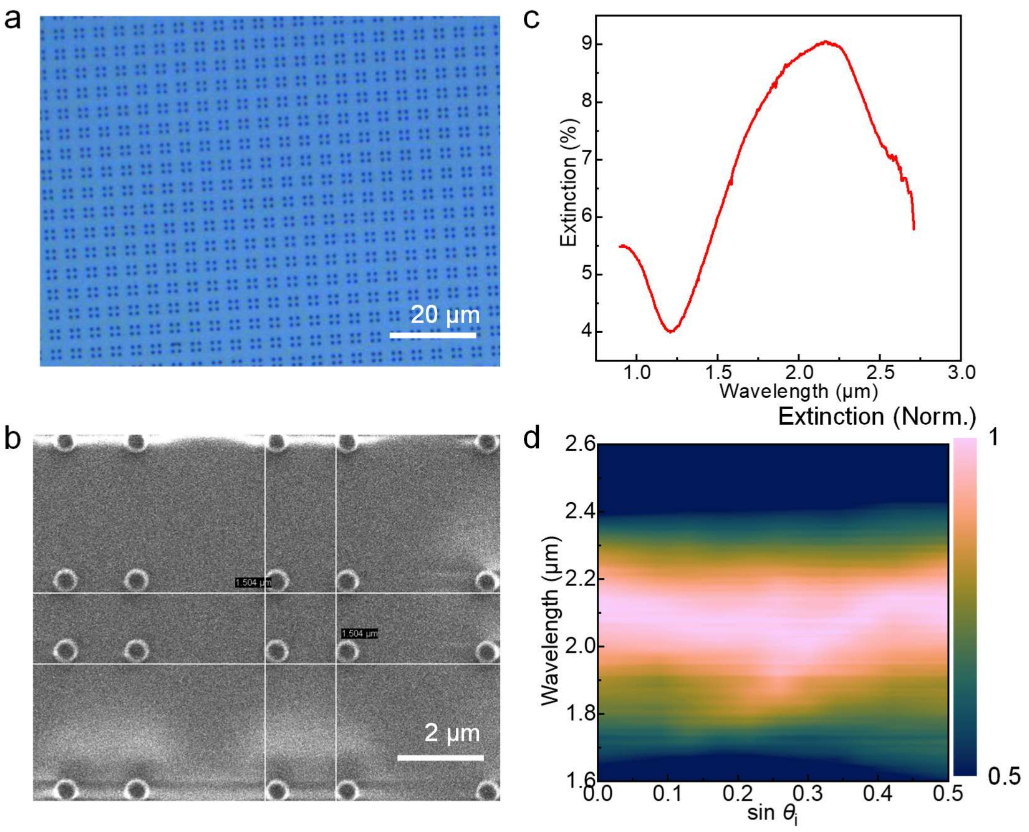

Supplementary Fig. 41 | Structures of 2 × 2 PEDOT nanoantenna matrix and its extinction spectra. (a) (b) Images observed by optical microscopy and SEM, respectively. (c) Experimental extinction

spectra under the normal radiation. (d) Angle-dependent extinction spectra (normalized) from experiments, based on p-polarization. The end-to-end distance of the PEDOT nanoantennas in the matrix is r = 1.5 μm, and the effective distance between neighboring nanoantennas belonging to two different matrix regions was set to $2r$ = 3.0 μm. Each nanoantenna has a diameter of 0.5 μm and a height of 0.2 μm. Such matrix exhibits a weak and broad resonance peak at 2.15 μm (linewidth FWHM ≈ 1.05 μm and quality factor $Q \approx 2$). Moreover, angle-dependent extinction spectra verify the non-dispersive feature in this resonance peak. Thus, only with the closest neighboring effect from adjacent nanoantennas, the surface plasmon resonance from 2 × 2 matrix still belongs to the localized mode.

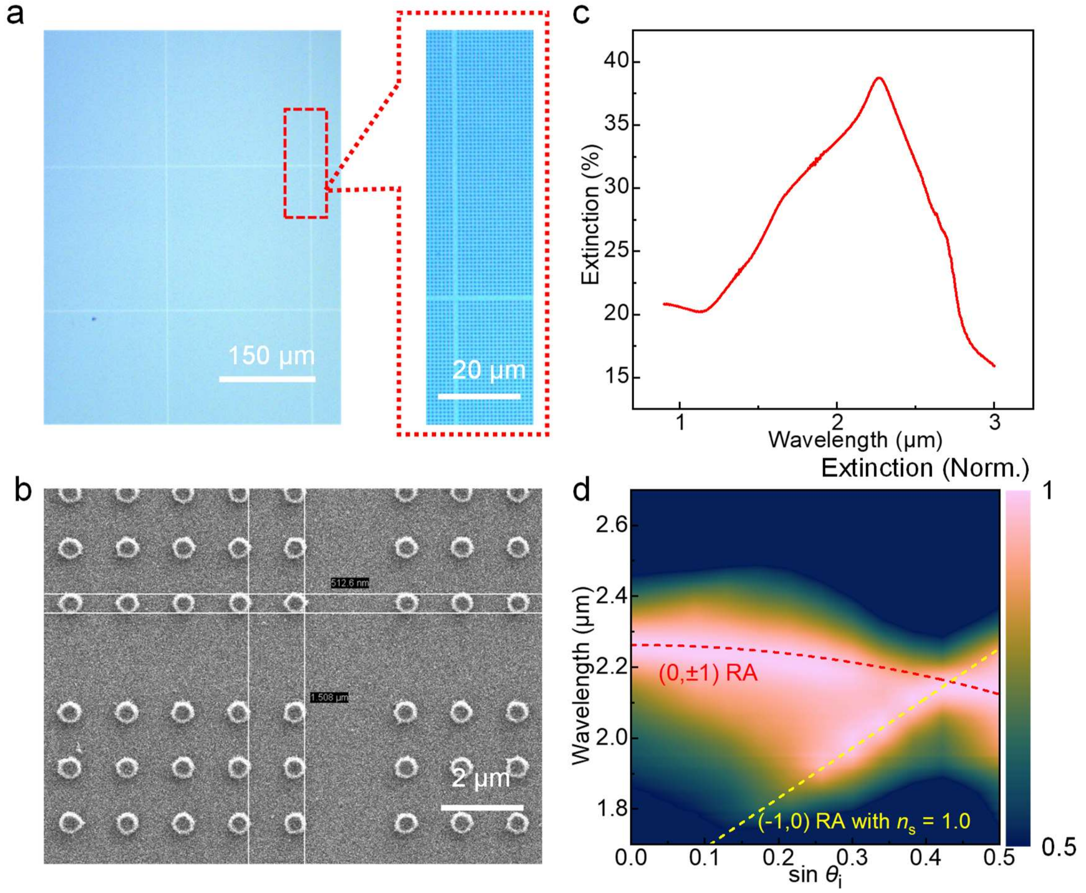

Supplementary Fig. 42 | Structures of 156 × 156 PEDOT nanoantenna matrix and its extinction spectra. (a) (b) Images observed by optical microscopy and SEM, respectively. (c) Experimental extinction spectra under the normal radiation. (d) Angle-dependent extinction spectra (normalized) from experiments, based on p-polarization. The end-to-end distance of PEDOT nanoantenna in the matrix is r = 1.5 μm, and the effective distance between neighboring nanoantennas belonging to two different matrix regions was set to $2r$ = 3.0 μm. Each nanoantenna has a diameter of 0.5 μm and a height of 0.2 μm. The extinction spectrum exhibits a narrower resonance peak at 2.26 μm. The angle-dependent extinction spectra illustrate that such resonance is efficiently coupled with (0, ±1) RA, confirming the presence of intense dipolar radiation. These observations verify the nonlocal resonance feature from the

156 × 156 matrix, which is almost the same as those in CLR features. Therefore, the sizes of ~230 μm ($N \approx 150$) can be sufficient to achieve the efficient benefit from CLRs.

Supplementary Note 5. Permittivity in the reduced state

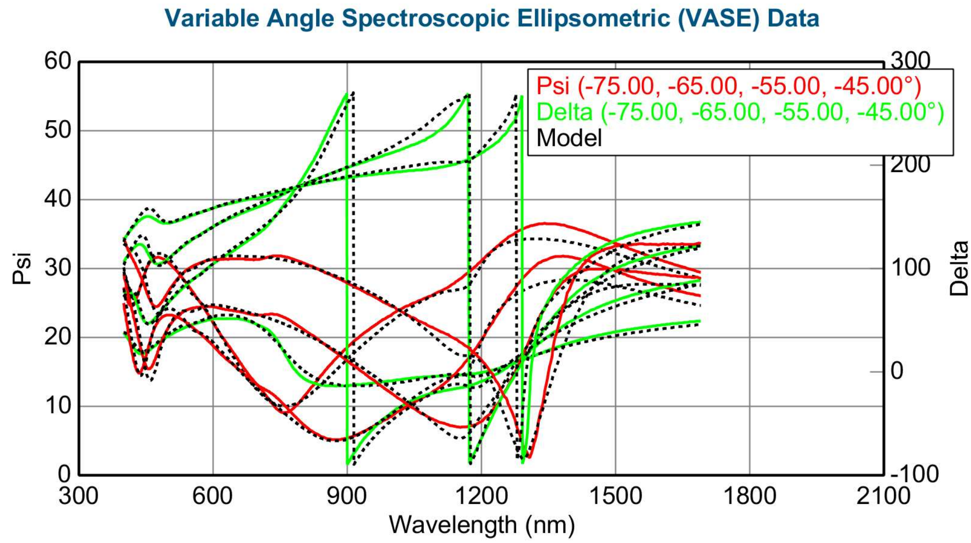

Supplementary Fig. 43 | Spectroscopic ellipsometry data (ranging from 210 nm to 1690 nm) for a PEDOT film in the reduced state (achieved by the PEI-treatment of acid-treated PEDOT:ToS). These raw data and the fitting were processed using the VASE software. The Psi (ψ, marked in the red line) and Delta (Δ, marked in the green line) were acquired at four angles (45°, 55°, 65°, and, 75°). The black dashed lines are the best fitting data by using the Drude-Lorents model.

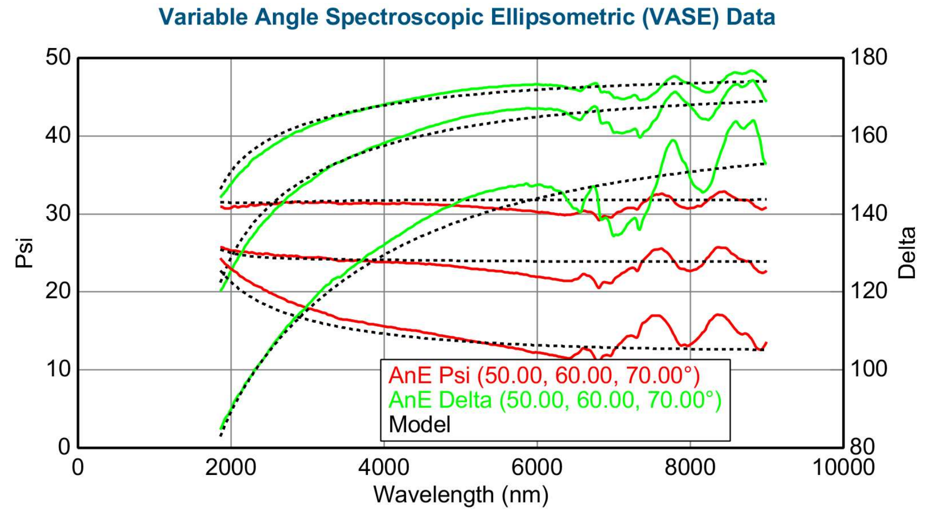

Supplementary Fig. 44 | Spectroscopic ellipsometry data (ranging from 1690 nm to 9000 nm) for a PEDOT film in the reduced state (achieved by the PEI-treatment of acid-treated PEDOT:ToS). These raw data and the fitting were processed using the VASE software. The Psi (ψ, marked in the red line) and Delta (Δ, marked in the green line) were acquired at four angles (45°,

55° , 65° , and, 75°). The black dashed lines are the best fitting data by using the Drude-Lorents model.

Supplementary Table 3: Oscillators for reduced state in the in-plane direction

ε_∞ = 1.100			
Oscillator No. (j th)	Frequency ω_j (eV)	Broadening γ_j (eV)	Amplitude A_j (eV^2)
Drude	0	0.449	0.149
1	2.018	0.766	4.700
2	6.684	1.494	48.984

Supplementary Table 4: Oscillators for reduced state in the out-of-plane direction

ε_∞ = 1.201			
Oscillator No. (j th)	Frequency ω_j (eV)	Broadening γ_j (eV)	Amplitude A_j (eV^2)
Drude	0	0.329	0.0428
1	4.199	0.213	17.65
2	2.195	0.227	0.353
3	1.036	0.0749	0.0354

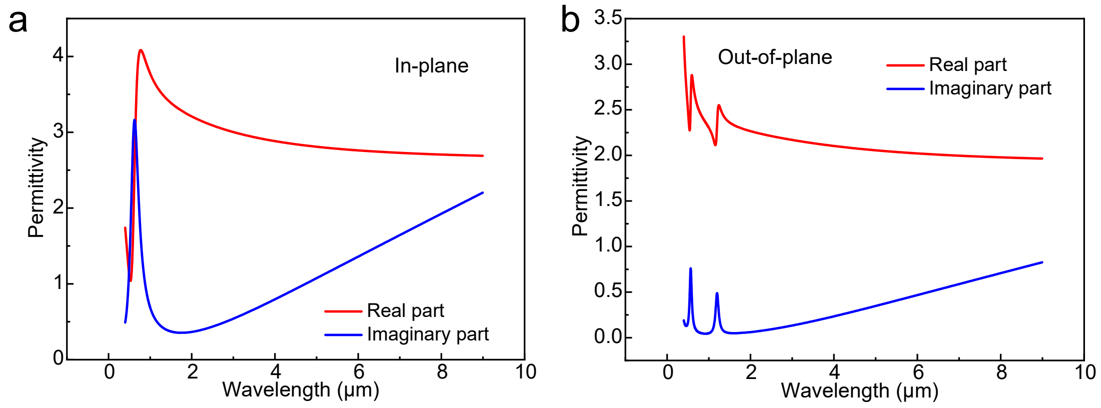

Supplementary Fig. 45 | Permittivity of PEDOT film in the reduced state (after PEI treatment and washed by DI water). These data are obtained from the calculation of Drude-Lorentz model by fitting the data of spectroscopic ellipsometry above. The film thickness was around 200 nm. (a) Along the in-plane direction. (b) Along the out-of-plane direction.

Supplementary Note 6. Redox recycles

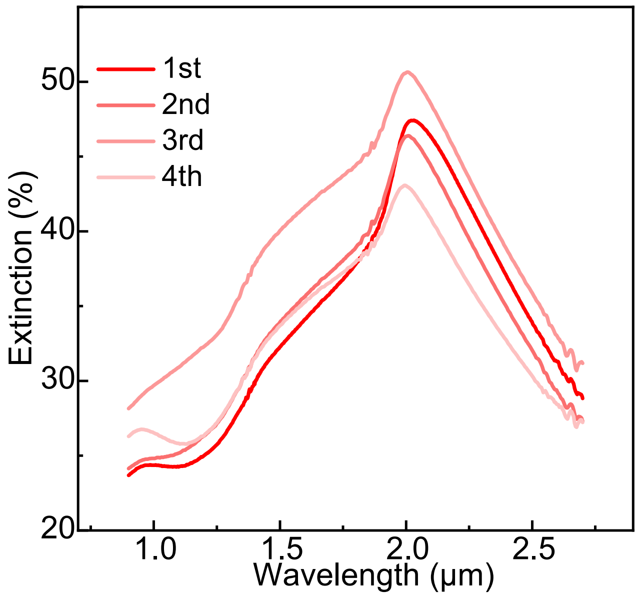

Supplementary Fig. 46 | Extinction spectra of collective lattice resonance after 1-4 redox cycles. The diameter of nanodisks was 0.50 μm, and the height of nanodisks was 0.2 μm. The periodic distance is 1.3 μm.

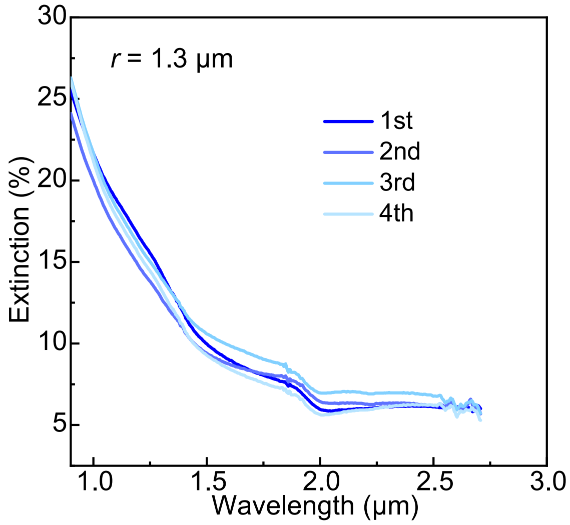

Supplementary Fig. 47 | Extinction spectra of reduced PEDOT nanoantennas after 1-4 redox cycles. The diameter of nanodisks was 0.50 μm, and the height of nanodisks was 0.2 μm. The periodic distance is 1.3 μm.

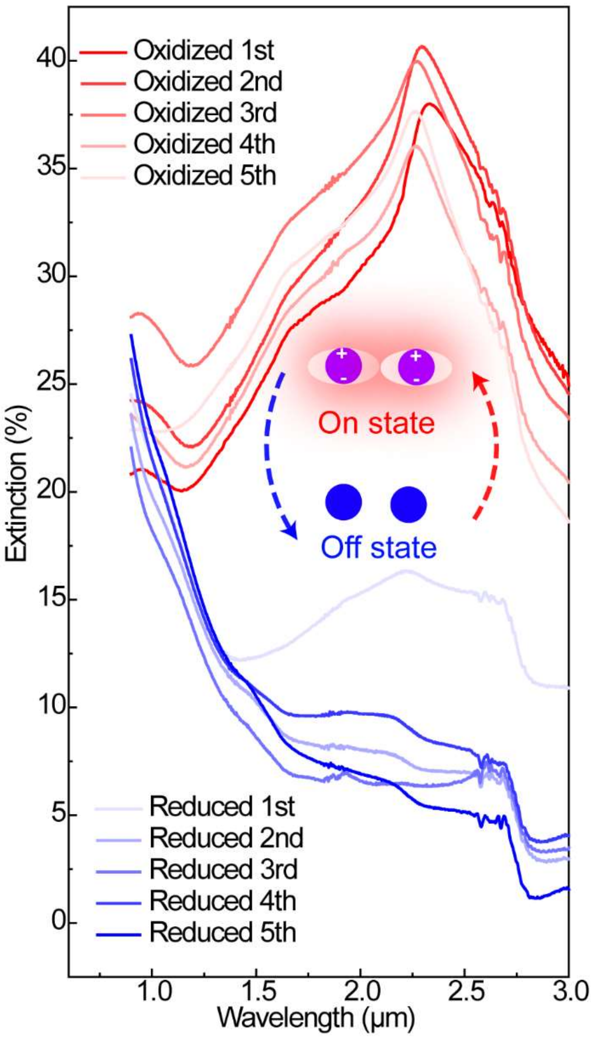

Supplementary Fig. 48 | Redox cycle performances of PEDOT-based periodic arrays with *r* = 1.5 μm. The diameter of nanodisks was 0.54 μm, and the height of nanodisks was 0.2 μm.

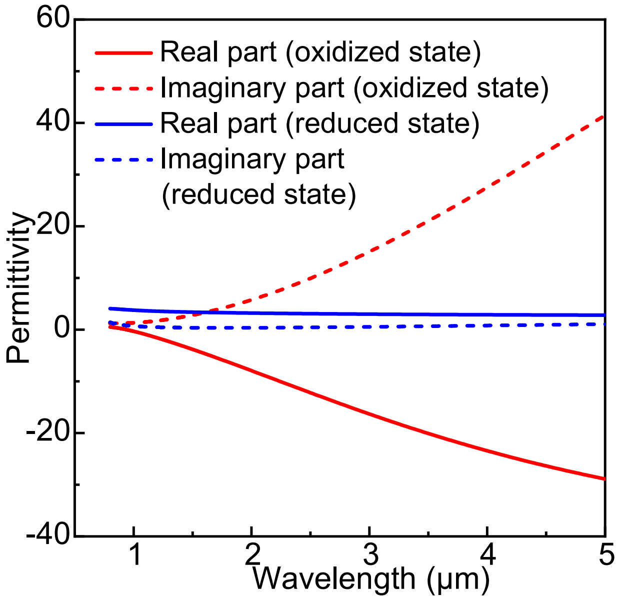

Supplementary Fig. 49 | In-plane permittivity dispersion of PEDOT films in different redox states. These data are extracted from Supplementary Fig. S3 and S40 in the wavelength region of 0.8-5 μm. The film thicknesses were about 0.2 μm.

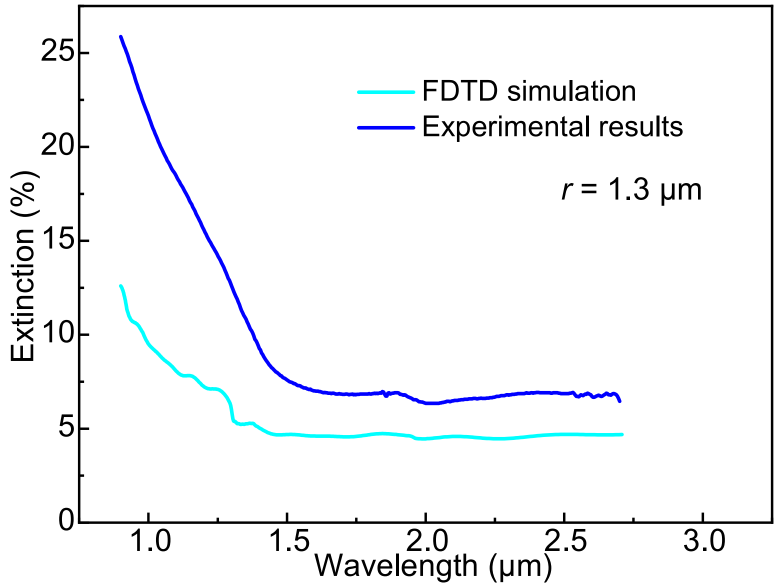

Supplementary Fig. 50 | Extinction spectra of PEDOT-based periodic arrays (*r* = 1.3 μm) in the reductive state. In both FDTD simulations and experimental results, the diameter of nanodisks was 0.5 μm, and the height of nanodisks was 0.2 μm.

Reference:

1. Chen S*, et al.* Conductive polymer nanoantennas for dynamic organic plasmonics. *Nat. Nanotechnol.* **15**, 35-40 (2020).

2. Humphrey AD, Barnes WL. Plasmonic surface lattice resonances on arrays of different lattice symmetry. *Phys. Rev. B* **90**, 075404 (2014).

3. Auguié B, Barnes WL. Collective Resonances in Gold Nanoparticle Arrays. *Phys. Rev. Lett.* **101**, 143902 (2008).

4. Liang Y, Tsai DP, Kivshar Y. From Local to Nonlocal High-Q Plasmonic Metasurfaces. *Phys. Rev. Lett.* **133**, 053801 (2024).

5. Huttunen MJ, Dolgaleva K, Törmä P, Boyd RW. Ultra-strong polarization dependence of surface lattice resonances with out-of-plane plasmon oscillations. *Opt Express* **24**, 28279-28289 (2016).

6. Guo R, Hakala TK, Törmä P. Geometry dependence of surface lattice resonances in plasmonic nanoparticle arrays. *Phys. Rev. B* **95**, 155423 (2017).
